# A Novel Pak1 Activator Ameliorates ER Stress for HFpEF Therapy

**DOI:** 10.1002/advs.76964

**Published:** 2026-08-03

**Authors:** Honglin Xu, Hongyuan Zhang, Tayyiba Azam, Susanne S. Hille, Yingjuan Liu, Min Zi, Jiaqing Lang, He Yu, Yujia Cao, Bernard D. Keavney, Tamer M. A. Mohamed, Jessica M. Miller, Riham R. E. Abouleisa, Ming Xu, Elizabeth J. Cartwright, Oliver J. Müller, Ming Lei, Xin Wang

**Affiliations:** ^1^ Division of Cardiovascular Science, Faculty of Biology, Medicine and Health The University of Manchester Manchester UK; ^2^ Department of Internal Medicine III University of Kiel Kiel Germany; ^3^ German Center for Cardiovascular Research (DZHK) Partner Site Hamburg/Kiel/Luebeck Hamburg Germany; ^4^ Division of Molecular and Cellular Function, School of Biological Sciences The University of Manchester Manchester UK; ^5^ Department of Pharmacology University of Oxford Oxford UK; ^6^ Department of Surgery Baylor College of Medicine Houston USA; ^7^ Department of Clinical Pharmacy, School of Preclinical Medicine and Clinical Pharmacy China Pharmaceutical University Nanjing China

**Keywords:** atf6, cardiac function curve, cell biology, endoplasmic reticulum, gene knockdown, heart failure with preserved ejection fraction, homeostasis, regulator, unfolded protein response

## Abstract

Heart failure with preserved ejection fraction (HFpEF) is a prevalent and complex syndrome, with metabolic dysfunction playing a pivotal role in its progression. Disrupted endoplasmic reticulum (ER) homeostasis is recognized as a central mechanism in its pathogenesis. Although the homologous kinases Pak1 and Pak2 regulate the ER stress response, the role of Pak1 in HFpEF remains unclear. This study demonstrates that Pak1 is a critical regulator of cardiac adaptation to metabolic stress. Using a murine HFpEF model combining high‑fat diet and nitric oxide synthase inhibition, Pak1 knockdown accelerates diastolic dysfunction and maladaptive remodeling, accompanied by disrupted ER ultrastructure and impaired PERK‑ATF4 signaling, whereas Pak1 overexpression preserves cardiac function and ER homeostasis. Mechanistically, Pak1 activates the ERK1/2‐MNK1‐eIF4E signaling axis and promotes adaptive integrated stress response (ISR) signaling through the PERK‐ATF4 pathway. Pharmacological inhibition of MNK1 attenuated Pak1‐mediated PERK activation and ATF4 induction, identifying a mechanistic link between Pak1 signaling and adaptive stress responses. Furthermore, we developed a novel small‐molecule Pak1 activator, JB2019, which reversed metabolic stress‐induced cardiac dysfunction in both HFpEF mice and cardiac organoids. Collectively, these findings identify Pak1 as a novel regulator of adaptive ISR signaling and establish Pak1 activation as a promising therapeutic strategy for HFpEF.

## Graphical abstract

Chronic metabolic stress is a major contributor to HFpEF progression. Under prolonged metabolic stress, Pak1 activity becomes impaired, contributing to disrupted ER proteostasis, cardiomyocyte apoptosis, fibrosis, and diastolic dysfunction. Mechanistically, Pak1 overexpression activates the ERK1/2–MNK1–eIF4E signaling axis, promotes translational adaptation, and engages adaptive PERK‐dependent integrated stress response signaling. Pharmacological activation of Pak1 restores ER homeostasis and improves cardiac outcomes in both HFD+L‐NAME‐induced HFpEF mice and PA‐stressed cardiac organoids, reducing fibrosis, improving diastolic function, and enhancing organoid beating activity.

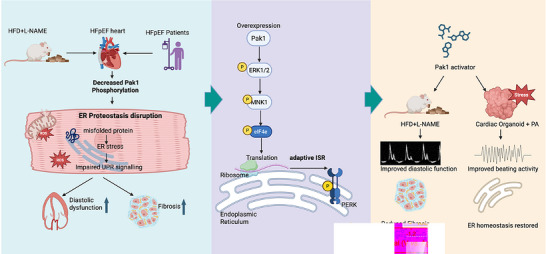

**Abbreviations**
AAV9: adeno‐associated virus serotype 9
ATF4: activating transcription factor 4
CHOP C/EBP: homologous protein
ER: endoplasmic reticulum
HFD: high‐fat diet
HFpEF: heart failure with preserved ejection fraction
hESCs: human embryonic stem cells
ISR: integrated stress response
IRE1: inositol‐requiring enzyme 1
IVRT: isovolumic relaxation time
L‐NAME: Nω‐Nitro‐l‐arginine methyl ester
PA: palmitic acid
Pak1: p21‐activated kinase 1
ROS: reactive oxygen species
UPR: unfolded protein response
XBP1s: X‐box binding protein 1 splicing.

## 1 Introduction

Heart failure with preserved ejection fraction (HFpEF) is a chronic, progressive cardiac syndrome closely linked to aging and a constellation of cardiometabolic risk factors such as hypertension, obesity, insulin resistance, and type 2 diabetes [1, 2, 3]. Unlike heart failure with reduced ejection fraction (HFrEF), HFpEF is marked by preserved ejection fraction with impaired diastolic function, often accompanied by impaired endoplasmic reticulum (ER) stress response, aberrant autophagy, mitochondrial dysfunction, low‐grade inflammation, oxidative stress, and impaired calcium handling [4, 5]. In particular, clinical data and experimental evidence revealed that the accumulation of misfolded proteins and the IRE1/XBP1 pathway inactivated occur in the HFpEF hearts [6, 7, 8], suggesting that, unlike acute infarction damage, hemodynamic/metabolic insult may impair ER function in a chronic manner that causes disturbances in cellular homeostasis and metabolic dysfunction attributable to HFpEF initiation and progression. Despite its rising prevalence and clinical burden, the underlying molecular mechanisms remain incompletely understood.

Chronic metabolic stress and the resultant disruption of cellular homeostasis play pivotal roles in HFpEF pathogenesis [7, 8, 9]. Central to this is the ER, an essential organelle responsible for protein folding, calcium handling, and lipid biosynthesis [10, 11, 12]. The proximity of the ER to the nucleus gives the ER unique control over gene expression and protein maturation that empowers it to significantly influence other organelle integrity and functions. The ER serves as a sentinel of intracellular homeostasis, mounting a protective response when faced with protein misfolding or metabolic overload, a process known as the unfolded protein response (UPR) [13, 14].

The UPR is orchestrated through three major signaling pathways, each governed by a key transcription factor: activating transcription factor 4 (ATF4), spliced XBP1 (XBP1s), and ATF6 [15]. These arms coordinate a cellular defense mechanism aimed at restoring ER function; however, sustained or excessive ER stress can lead to maladaptive responses, including apoptosis and calcium imbalance, which contribute to cardiac dysfunction and adverse remodeling in HFpEF [16, 17]. While previous studies have implicated ER stress in cardiac diseases [18, 19], how it specifically shapes the HFpEF phenotype, and which branches of the UPR are most critical, remains poorly defined.

The p21‐activated kinase 1 (Pak1) has emerged as a potential regulator of cardiac homeostasis [20, 21]. Pak1 is a serine/threonine kinase involved in a wide range of cellular processes, including cytoskeletal organization, survival signaling, and stress responses [22, 23]. Previous investigations from us and others have demonstrated that Pak1 and Pak2 protect the heart from various types of stress. Pak2 is abundantly localized near the ER membrane and acts as a regulator of the IRE1/XBP1‐dependent UPR for cardioprotection. Cardiac deficiency of Pak1 was thus shown to contribute to tachyarrhythmia following isoprenaline (ISO) stimulation [24]. In addition, Pak1 mitigates cardiac hypertrophic remodeling by antagonizing the mitogen‐activated protein kinase (MAPK) pathway [25]. Reduced Pak1 activity has also been associated with increased production of reactive oxygen species (ROS) [26]. Although Pak1 and Pak2 share 91% sequence identity, the role of Pak1 in ER stress response and the progression of HFpEF remains to be fully elucidated. In this study, we investigated the role of Pak1 in promoting UPR in defending HFpEF, with a particular focus on the ATF4, XBP1s, and ATF6 pathways. By combining in vivo and in vitro models, we aimed to delineate the molecular interplay between Pak1 signaling and ER stress response. We also evaluated the therapeutic potential of a novel small molecule Pak1 activator [27, 28, 29], JB2019, which binds to a site within the auto‐inhibitory domain (AID) of Pak1 and induces an allosteric conformational change in Pak1, stabilizing its active form. This Pak1 activator worked as a strategy to restore ER homeostasis and improve diastolic function in HFpEF.

## 2 Methods

### 2.1 Animal Models

All animal experiments were conducted in strict accordance with the United Kingdom Animals (Scientific Procedures) Act 1986 and approved by the University of Manchester Ethics Committee under project licenses P3A97F3D1 and PP5982529. C57/BL6N male mice were maintained under specific pathogen‐free conditions with a controlled 12‐h light/dark cycle. Since previous research suggests that female mice may exhibit different responses to metabolic and hypertensive stressors, the study primarily utilized young male mice, despite HFpEF being an age‐ and sex‐related disease [30, 31]. For biochemical, histological, cardiac function, and echocardiographic analyses, groups of 5 to 8 animals were utilized. All in vivo procedures were performed under blinded conditions with respect to treatment during both data acquisition and analysis. Euthanasia was carried out by cervical dislocation followed by immediate cardiac excision prior to the irreversible cessation of circulation. All experiments were conducted in strict accordance with the guidelines of the European Union Directive 2010/63/EU on the protection of animals used for scientific purposes.

### 2.2 2‐Hit HFpEF Model

To model the comorbidities observed in human HFpEF, a two‐hit strategy was employed in C57BL/6N male mice, combining a high‐fat diet (HFD) with administration of the constitutive nitric oxide synthase inhibitor Nω‐nitro‐L‐arginine methyl ester (L‐NAME; Sigma‐Aldrich) at a concentration of 0.6 g/L in drinking water to induce hypertension and metabolic stress [32]. Drinking water containing L‐NAME was refreshed every 3 days. Beginning at 6–8weeks of age, mice were randomized to receive either the HFD or standard chow diet. The HFD formulation (824054, Special Diets Services) consisted of 60% fat, 20% protein, and 20% carbohydrate.

### 2.3 Human Heart Sample Collection

Fresh human hearts were provided by consented donors through the USA Transplantation Network. All procedures were in agreement with the Declaration of Helsinki and were approved by the Institutional Review Boards (IRBs) of Baylor College of Medicine (IRB protocol no. H5326).

Normal healthy donors inclusion criteria: 1) age greater than or equal to 50, 2) capable of giving informed consent, and 3) ejection fraction >55%. Exclusion criteria: 1) history of myocardial infarction, 2) pregnant women, 3) decompensated heart failure, 4) patients with severe kidney disease, 5) asthma or severe chronic lung disease, 6) cardiac pacemaker or implantable defibrillator, 7) cerebral aneurysm clip, 8) neural stimulator, and 9) history of diabetes.

HFpEF donors inclusion criteria: 1) age greater than or equal to 50, 2) capable of giving informed consent, 3) ejection fraction >55%, 4) impaired relaxation, 5) patients with chronic kidney disease, 6) history of hypertension, and 7) history of diabetes. Exclusion criteria: 1) history of myocardial infarction, 2) pregnant women, 3) decompensated heart failure, 4) asthma or severe chronic lung disease, 5) cerebral aneurysm clip, and 6) neural stimulator.

### 2.4 Adeno‐Associated Virus 9 (AAV9) Construction and Injection

AAV9‐TnT‐shPak1‐miR30hp‐mCherry was used for Pak1 knockdown, and AAV9‐TnT‐shctrl‐miR30hp‐mCherry was used as control. The shPak1 (Horizen) sequences targeting CDS region of mice Pak1 (Primer sequence: GCATTAAAGCAGCGTATC). The sequences were then cloned into a pSSV9‐TnT‐shRNA‐miR30hp‐EGFP vector under a cardiac‐specific promoter cTNT and packaged into AAV9‐shPak1. The vector plasmid provided by Professor Oliver J. Müller (University of Kiel) has been previously validated for its effectiveness. The AAV9‐Pak1‐T403E was used for Pak1 overexpression in cardiomyocytes. In brief, the pSSV9‐TnT‐eGFP plasmid was edited by replacing GFP with Flag‐tagged constitutively active Pak1 (T403E) cDNA (Addgene) to construct the pSSV9‐TnT‐Pak1 plasmid, then packaged into AAV9‐Pak1, AAV9‐GFP was used as the control. To establish the experimental knockdown model, mice first received intravenous injection of 1 × 10^11^ genomic virus particles via the tail vein for 2 weeks, after which HFD and L‐NAME treatment was initiated. The overexpression model is to start feeding with fat one week after virus injection. The body weight of the mice was monitored weekly. Pak1 knockdown and overexpression were confirmed by western blot at the end of the experiments.

### 2.5 Echocardiography

Echocardiographic assessments were performed to examine cardiac structure and function. Mice underwent chest hair removal by applying depilatory cream for 10–15 s, followed by cleansing with a wet tissue to ensure complete hair removal. Anesthesia was induced with 1.5% isoflurane and maintained at 0.5%–1% during imaging, ensuring the heart rate remained above 450 beats per minute. Cardiac imaging was acquired using a Vevo 3100 Preclinical Imaging System equipped with a 30‐MHz transducer (FUJIFILM VisualSonics, Canada). Data were analyzed using Vevo Lab 5.6.1 (FUJIFILM VisualSonics, Canada).

### 2.6 Lung Ultrasound

Lung ultrasound imaging was performed along the midaxillary line, with two‐dimensional images captured for analysis. Under normal conditions, the lungs appear as a black region on ultrasound, characterized by a thin pleural line accompanied by multiple parallel hyperechoic A‐lines. In cases of reduced pulmonary air content and increased lung density, such as with pulmonary edema, inflammation, or tissue remodeling, ultrasound waves generate B‐lines, which are distinct, laser‐like vertical hyperechoic artifacts arising from the pleural line and extending to the bottom of the image without attenuation. Additional abnormal findings include the absence of A‐lines, increased overall lung echogenicity (whiteness), and thickening of the pleural line. A modified lung ultrasound scoring system, based on Villalba‐Orero et al. [33] was applied to assess lung injury severity, evaluating the presence of B‐lines (scored 0–2), absence of A‐lines (0–2), lung whiteness (0–2), and pleural line thickening (0–1). The cumulative score ranged from 0 (normal) to 7 (severe injury). B‐line scores were assigned based on the number and confluence of vertical artifacts observed within each imaging field.

### 2.7 Blood Pressure

Blood pressure in conscious mice was measured using a non‐invasive tail‐cuff system (CODA, Kent Scientific Corporation, USA). Cuff pressure was inflated to 250 mmHg to occlude tail blood flow, then gradually released over 15 s. Each session included 20 cycles, with the first 10 cycles discarded for acclimation. Only measurements with a tail blood volume >15 µl were included. Systolic and diastolic pressures were automatically calculated during cuff deflation. At least five valid readings were averaged per mouse. Mice were acclimated to the restraining tube over 3 days before baseline measurements. All recordings were performed at the same time each day in a quiet room to minimize variability.

### 2.8 Intraperitoneal Glucose Tolerance Test

Mice were fasted for 6 h in a clean cage with free access to water prior to testing. Body weight and fasting blood glucose levels were recorded before administering a 20% glucose solution intraperitoneally at a dose of 2 g/kg body weight. Following glucose injection, blood samples were collected from the tail vein at 15, 30, 45, 60 min, and 2 h post‐injection. Blood glucose levels were measured at each time point using an Accu‐Chek Aviva glucometer (Roche).

### 2.9 Wheat Germ Agglutinin Staining

Cardiomyocyte cross‐sectional areas were quantified following wheat germ agglutinin (WGA) staining. Tissue sections were initially dewaxed in xylene for 1 h, rehydrated through graded ethanol solutions (100%, 75%, and 50%; 5 min each), and subsequently rinsed in double‐distilled water (ddH_2_O) for 5 min. The sections were stained with WGA (Texas Red‐X Conjugate in a dark humidified chamber at room temperature for 1 h. Next, the sections were washed 3 times with PBS for 10 min each before being mounted using Eukitt quick‐hardening medium (Fluka, 03989), and imaged using a snapshot widefield microscope. Quantitative analysis of cardiomyocyte cross‐sectional areas was performed using ImageJ software, with measurements obtained from 100 cardiomyocytes across three independent sections per mouse.

### 2.10 Masson's Trichrome Staining

Interstitial fibrosis was evaluated by Masson's trichrome staining. Tissue sections were initially dewaxed in xylene, followed by sequential rehydration through graded ethanol solutions (100%, 75%, and 50%) and distilled water, each for 5 min. Sections were subsequently incubated in Bouin's solution for a minimum of 4 h and rinsed under running tap water for 20 min. Nuclear staining was achieved using Harris’ hematoxylin solution for 5 min, followed by differentiation in 1% hydrochloric acid in 70% ethanol for 10 s. Sections were then washed under running warm water for 5 min until clear blue nuclei were observed. Subsequently, sections were stained with Biebrich Scarlet‐Acid Fuchsin solution for 5 min, treated with 2.5% (w/v) phosphomolybdic acid for 20–25 min, and counterstained with Light Green‐Acetic Acid solution for 3 min. Dehydration was performed through three changes of 100% industrial methylated spirit (IMS) for 5 min each, followed by clearing in xylene for 20 min. Finally, sections were mounted using Eukitt quick‐hardening mounting medium. Imaging the left ventricular sections was performed using a Zeiss Axioplan2 microscope, with fibrotic areas identified by blue staining. The extent of interstitial fibrosis was quantified using ImageJ software.

### 2.11 Dihydroethidium Staining

Superoxide production in cardiomyocytes was assessed in situ using the oxidative fluorescent dye dihydroethidium. The tissue sections were first dewaxed and rehydrated, then incubated for 30 min at 37°C in a dark, humid chamber with dihydroethidium (20 µm in PBS; Invitrogen). Fluorescent images were captured using a Zeiss Axioplan2 microscope, and the resulting data were analyzed with ImageJ software.

### 2.12 Transmission Electron Microscope

Subcellular organelle morphology was examined using transmission electron microscopy (TEM). A 1 × 1 mm^3^ fragment of cardiac tissue was excised immediately after euthanasia and fixed in 0.1 m HEPES buffer (pH 7.2) containing 4% formaldehyde and 2.5% glutaraldehyde. Samples were subsequently treated with 0.1 M cacodylate buffer (pH 7.2) supplemented with 1.5% potassium ferrocyanide and 0.1% osmium tetroxide for 1 h, followed by incubation in 0.1 M cacodylate buffer containing 1% uranyl acetate for 1 h, and then in 1% uranyl acetate alone for an additional hour. Dehydration was carried out through a graded ethanol series, and tissues were embedded in TAAB 812 resin and polymerized at 60°C for 24 h. Ultrathin sections were obtained using a Reichert Ultracut ultramicrotome (Reichert‐Jung, Vienna, Austria) and imaged with a Talos L120C transmission electron microscope (Thermo Fisher Scientific, USA) operating at 100 kV.

### 2.13 Cell Culture

Human ventricular cardiomyocutes, AC16 cells were cultured in Dukbecco's Modified Eagle Medium (DMEM)/Nutrient Mixture F‐12 (Gibco, 31330), supplemented with 10% fetal bovine serum (FBS) and 1% penicillin‐streptomycin. H9c2 cells were cultured in DMEM (Gibco, 41965) Cells were maintained in a humidified chamber at 37°C with 5% CO_2_ and were passaged upon reaching approximately 80%–90% confluence using trypsin. Culture medium was refreshed every 2–3 days.

### 2.14 Primary Mouse Cardiomyocyte Isolation and Culture

Primary adult mouse cardiomyocytes were isolated by retrograde Langendorff perfusion. Briefly, mice were anesthetized, and hearts were rapidly excised and mounted onto a Langendorff perfusion system. Hearts were first perfused with Ca^2+^‐free perfusion buffer containing NaCl 134 mm, HEPES 10 mm, NaH_2_PO_4_ 1.2 mm, KCl 4 mm, MgSO_4_ 1.2 mm, and glucose 11.1 mm (pH to 7.3‐7.4 adjusted with NaOH) to remove blood and equilibrate the myocardium. The hearts were then digested with isolation solution supplemented with collagenase I (C0130, Sigma‐Aldrich, USA) and protease (P5147, Sigma‐Aldrich) until the tissue became soft and pale. Ventricular tissue was gently minced and dissociated by repeated gentle trituration. The resulting cell suspension was filtered through a 200 µm nylon mesh filter. Calcium was gradually reintroduced to the isolated cardiomyocytes to restore physiological calcium tolerance. Cells were then plated on culture dishes maintained in culture medium under standard conditions at 37°C with 5% CO_2_. For palmitic acid stimulation, freshly isolated cardiomyocytes were allowed to recover for 1 h before treatment with 500 µm palmitic acid, followed by protein extraction for western blot analysis.

### 2.15 Induction of Metabolic Stress In Vitro

To investigate the impact of metabolic stress on cardiomyocytes, AC16 or mice cardiomyocytes cells were treated with 500 µm palmitic acid (PA; Sigma) conjugated to 10% fatty acid‐free BSA. Before PA treatment, cells were starved 2 h in serum‐free DMEM to normalize kinase activity to baseline levels.

### 2.16 Small Interfering RNA‐Mediated Knockdown

Lipofectamine 2000 Transfection Reagent (Invitrogen, cat 11668019) was applied to transfect siRNA (Thermo Fisher Scientific, s131401) for Pak1 knockdown into cardiomyocytes following the manual instructions. In brief, AC16 cells were washed with antibiotic‐free ACCT medium twice before being treated with siRNA. siRNA and Lipofectamine 2000 were diluted in Opti‐MEM I Reduced Serum Medium (Invitrogen, cat 31985062), respectively, for 5 min at room temperature, and then were mixed to incubate for 25 min before being added to each well‐containing cell and medium. The medium was normally changed after 6 h of incubation to allow efficient siRNA transfection. The cells were kept in the incubator for 1 or 2 days before analysis of gene expression or any other experimental treatment.

### 2.17 MTT Cell Viability Assay

Cytotoxicity was estimated by MTT colorimetric assay. 5 × 104 cells/well in 100 µL culture medium were seeded in wells of a 96‐well plate. Following different concentrations of PA treatment for 12 h, the media was discarded, and 100 µL MTT solution (5 mg/mL in PBS) was added to each well alongside 100 µL serum‐free DMEM media (Gibco). Cells were incubated for 3 h at 37°C before the solution was removed and the wells left to dry overnight at room temperature in the dark. 150 µL DMSO was to each well as an MTT solvent, and the plate shaken for 15 min at room temperature whilst covered in foil to prevent light affecting the colorimetric results. Absorbance was measured on a Synergy HT microplate reader (Promega) at a wavelength of 570 nm, with the background subtracted at 630 nm. Cell viability was expressed as a percentage of untreated controls.

### 2.18 Adenoviral Production and Infection

To prepare the recombinant adenovirus that expresses constitutively active Pak1 (AdPak1), the polymerase chain reaction method was used to tag the human Pak1 cDNA with an HA epitope (YPYDVPDYA) at the N‐terminal region and next to the translational initiation codon. Threonine 423 was mutated to glutamic acid in Pak1 cDNA to convert the Pak1 protein into a constitutively active form. The cDNA was cloned into a shuttle vector pAdCMV to obtain pAdCMVPak1. The AdPak1 was made by homologous recombination between pAdCMVPak1 and the viral backbone DNA DL7001. Subsequently, viral amplification and purification were carried out following established protocols [34]. In summary, human embryonic kidney 293T cells were transfected with the purified plasmids and collected once approximately 80% cytopathic effect was evident. The cells underwent three freeze‐thaw cycles to release viral particles, followed by gradient centrifugation using caesium chloride (densities of 1.33 and 1.45 g/mL). The adenovirus‐containing band was extracted and further purified via dialysis tubing (pore size, 24 Angstrom; Medicell). The adenovirus was then used to infect cells at a multiplicity of infection (MOI) of 12.5.

### 2.19 Puromycin Incorporation Assay

Protein synthesis was assessed using the puromycin assay. AC16 cells were incubated with puromycin (A1113803, Gibco Puromycin Dihydrochloride) (10 µg/mL) for 10 mins at 37°C to allow incorporation into newly synthesized polypeptides. Cells were then washed twice with ice‐cold PBS and lysed in lysis buffer containing protease and phosphatase inhibitors. Protein lysates were collected and subjected to Western blot analysis using an anti‐puromycin antibody (MABE343, Merck) to quantify global protein synthesis. For immunofluorescence‐based detection of protein synthesis, AC16 cells were cultured on glass coverslips and incubated with puromycin (10 µg/mL) for 10 min prior to fixation with 4% paraformaldehyde. Cells were permeabilized with 0.1% Triton X‐100, blocked with 5% bovine serum albumin (BSA), and incubated with an anti‐puromycin mouse antibody (MABE343, Merck). The endoplasmic reticulum (ER) was stained using an anti‐Calnexin rabbit antibody (2679S, Cell Signaling Technology), and nuclei were counterstained with DAPI. Secondary antibodies used were Alexa Fluor 568 donkey anti‐mouse IgG (A10037, Invitrogen) and Alexa Fluor 488 goat anti‐rabbit IgG (A11008, Invitrogen).

### 2.20 Cardiac Cells Differentiation

At the time of starting human embryonic stem cell‐derived cardiomyocytes (hESC‐CM) differentiation, hESCs (WA09 cell line, WiCell) should be at 90%–100% confluency, and the time is termed as Day 0. On Day 0 and Day 1, 6 µm CHIR99021 (Selleckchem) in B27‐ medium (RPMI 1640 with GlutMax (Gibco) supplemented with B27 minus insulin (A1895601, ThermoFisher) in a ratio of 49:1) was added onto hESCs for WNT activation. On Day 2–3, the medium was changed to B27^−^ with 2 µm C59 (Selleckchem) for WNT inhibition. From Day 4 to Day 8, the medium was changed into pure B27^−^ medium. Cell beatings typically started to be observed from Day 6–8. From Day 9 to Day 15, the medium was changed to B27^+^ medium (RPMI 1640 with GlutMax (Gibco) with 2% B27 supplement (Gibco)) for cardiomyocyte maintenance. For CM purification, B27^+^ without D‐Glucose and supplemented with 4 mm Lactate is added during Day 12–15.

For the hESC‐derived endothelial cells (EC), from day 0 to day 1, hESCs were cultured with B27^−^ medium containing 100 ng/ml AcivinA (R&D Systems). From day 1 to day 4, the medium was changed to B27^−^ medium with 10 ng/ml FGF (R&D Systems) and 10 ng/ml BMP4 (R&D Systems). From day 5 to day 10, the medium was changed to Endothelial Cell Growth Medium (ECM, PromoCell) with 100 ng/ml VEGF (Sigma‐Aldrich), ECM was applied till day 16.

For the hESC‐derived cardiac fibroblasts (CF), from day 0 to day 1, hESCs were cultured with B27 medium containing 12 µm CHIR99021 (Selleckchem). From day 1 to day 2, the medium was changed to B27^−^ medium only. From day 3, the medium was changed to Fibroblast Growth Medium (PromoCell) with 75 ng/ml bFGF (R&D Systems) till day 16.

### 2.21 Organoid Formation

Triculture organoids were created by combining hESC‐CMs with hESC‐ECs and hESC‐CFs in B27^+^ medium at a physiological cell ratio of 7:2:1 in 96‐well plates. The three types of cells were digested by adding 0.5 mL 1:1 mixture of TrypLE (10x) and Accutase to each well of the 12‐well plate, then they were put back to 37°C for 5–10 min (longer incubation time for EC samples about 15–20 min), DMEM/F12 medium with 5%KSR (knockout serum replacement) was added to stop digestion. The medium was transferred into a 15 mL tube and centrifuged at 1000 rpm for 10 min. After the supernatant was removed, B27^+^ medium was added to resuspend the cell pellets. The Countess II automatic cell counter was used for counting the cells. Each type of cell count is recorded, and cell concentrations were adjusted to 1 × 10^6^/mL (depending on the requirement of sizes). The cells were then mixed at the ratio of 7:2:1 (CM:EC:CF) in a new falcon tube. 40 ul cell mixture was aliquoted into each well of an ultra‐low attachment 96‐well plate for organoid composition. After 2 days, organoids were newly formed, and 160 µL organoid medium (B27^+^ medium with 20% ECM) was added into each well. Medium refresh with 100 µl every 2 or 3 days, then organoids were transferred to a low attachment 12‐well plate to get rid of debris after 2 weeks of co‐culture in ULA 96 well plate, and maintained for another 30 days before treatment.

### 2.22 Immunofluorescent Staining

The cells culture medium was removed and cells washed twice on ice cold PBS. Cells were fixed in 4% w/v PFA for 30 min at 4°C, and subsequently washed in PBS for 5 min, twice. Permeabilization was achieved through incubation in 0.1% v/v triton X‐100 and 0.1% Sodium Citrate for 8 min prior to blocking in 3% donkey serum for an hour at RT.

Primary antibody (diluted accordingly in 1% donkey serum) was added to each well and incubated overnight at 4°C. The next day, the primary antibody was removed, and the cells were washed in PBS for 5 min, 3 times, prior to the addition of the appropriate secondary antibody (diluted accordingly in 1% donkey serum) and incubated for an hour at RT. Cells were washed twice in PBS for 5 min, and subsequently subjected to 4',6‐diamidino‐2‐phenylindole (DAPI, Life Technologies; diluted at 1:1000 in 0.5% w/v BSA) for 5 min. The cells were further washed in PBS for 5 min, twice, prior to imaging. Fluorescent images were viewed using a snapshot microscope.

### 2.23 Calcium Imaging for Cardiac Organoids

The Fluo‐4 Calcium Imaging Kit (Thermo Fisher Scientific) used for the specific detection of calcium flux by imaging. Procedures were conducted following the manual protocol of the kit.

Fluo‐4 kit contains the ready‐to‐use dye solution. The live samples were washed once with warm PBS/B27 medium before staining with 1:1000 diluted Fluo‐4 dye in fresh B27 medium for 30 min in the incubator. After incubation, check the signal under EVOS FL imaging system. If an adequate GFP signal was observed, samples were subjected to at least two washes with warmed PBS and then returned to warm B27 medium in the incubator for another 30 min–1 h before imaging recording. The videos of stained organoid living samples were recorded with an EVOS imaging system connected to a video capture card and an OBS video recorder. Each record was kept at least 20s in duration and a frame rate of 60/s. The beating activity and calcium flux were analyzed with ImageJ. Normalized F/F0 was calculated as the peak calcium fluorescence (F) divided by the start fluorescence level (F_0_), then divided by the F/F_0_ of the baseline transient before treatment. Time to peak calcium (sec) was calculated as the difference in time between the peak calcium and the start of the calcium transient.

### 2.24 ER Tracker and ROS Imaging

Following similar procedures, organoid samples with and without chemical treatments were stained with CellROX Green (Thermo Fisher Scientific) and ER tracker (Thermo Fisher Scientific) according to the manufacturer's instructions to detect the ROS activities and ER distribution inside the cells. In brief, CellROX Green dye was added to the well of individual organoids (moved into 24well plate, one sample per well) at a final concentration of 5 µm and incubated for 30 min at 37°C, 5% CO2. Following incubation, organoids were washed twice with culture medium and then imaged with EVOS FL microscope. The procedure can also be applied to 2D monolayer cell cultures.

### 2.25 Contraction Analysis of Beating Spheroids

Videos of spontaneously beating spheroids from each group were recorded at 37°C for each condition using a Carl Zeiss Axiovert A1 Inverted Microscope and cellSENS Microscope Imaging Software (Olympus, Japan). Videos were converted to a series of TIFF format pictures by Adobe Premiere (Adobe, San Jose, CA). Threshold edge‐detecting in ImageJ software (National Institutes of Health) was used on high contrast spheroid picture series and graphed to realize beating profiles of fractional area change (i.e., contraction amplitude), from which beats per minute and contraction amplitude were calculated. Contraction amplitudes were calculated as the percent change in fractional area change amplitude between contraction and relaxation.

### 2.26 Compound Treatment

We developed a series of small‐molecule compounds that activate Pak1 and Pak2 (Small molecule Pak activators) (UK priority application, Ref: 439.483.128566) [27, 29]. The effect of JB2019 is examined to increase the activation of Pak1/2 with an EC50 of 8.6 µm by RapidFire mass spectrometry kinase assay. The JB2019 was dissolved in DMSO before treatment.

The effect of the JB2019 compound on HFpEF was investigated in vivo. Following 10‐weeks of the HFpEF regime, mice were treated with JB2019 at 10 mg/kg/day for 5 weeks via osmotic mini‐pumps (Alzet). The pumps were implanted subcutaneously in both Chow control mice and HFpEF mice. After mini‐pump installation, buprenorphine (0.1 mg/kg, subcutaneously) was administered daily in the following 3 days to reduce pain and suffering. Amounts required JB2019 were calculated based on the body weight of individual animals.

### 2.27 Quantitative Polymerase Chain Reaction

Total RNA was isolated from ventricular tissues or cultured cells using TRIzol reagent (Invitrogen, Carlsbad, CA, USA). One microgram of RNA was reverse transcribed into cDNA using the LunaScript RT SuperMix Kit (NEB#E3025). Quantitative PCR (qPCR) was conducted in triplicate using SYBR Select PCR Master Mix, following the manufacturer's protocol on a Step One Plus PCR System (Applied Biosystems). Gene expression levels were quantified using the 2^−ΔΔCT^ method, with Gapdh serving as the internal control for normalization.

### 2.28 Lysate Preparation and Western Blotting

Total protein was extracted from tissue samples using Triton lysis buffer composed of 20 mm Tris, 137 mm NaCl, 2 mm EDTA, 1% Triton X‐100, 25 mm β‐glycerophosphate, 1 mm Na_3_VO_4_, 1 mm PMSF, 1.54 µm aprotinin, 21.6 µm leupeptin, and 10% glycerol, adjusted to pH 7.4. Protein concentrations were measured using the Bio‐Rad protein assay. For Western blot analysis, 20 µg of protein per sample was loaded and probed with specific antibodies listed in Table S1.

### 2.29 Analysis of Single‐Nucleus RNA‐seq Data

Publicly available human myocardial single‐nucleus RNA sequencing (snRNA‐seq) data from patients with HFpEF and non‐failing controls were obtained from the study by Hahn et al. [35]. The original dataset was generated from human myocardial samples and processed as described in the publication [35]. In the present study, we performed a secondary analysis of the published snRNA‐seq dataset to assess pathway‐level transcriptional changes relevant to our experimental findings. Cardiomyocyte nuclei were identified based on the cell‐type annotations provided in the original dataset, and subsequent pathway analysis was restricted to the cardiomyocyte population. Gene set enrichment analysis (GSEA) was performed to determine whether the unfolded protein response pathway was enriched in HFpEF. The analysis was restricted to the predefined MSigDB Hallmark gene set “HALLMARK_UNFOLDED_PROTEIN_RESPONSE”. Enrichment was reported as the normalized enrichment score (NES), nominal *p* value, false discovery rate (FDR)‐adjusted q value.

### 2.30 Statistical Analysis

All data were expressed as mean ± SEM (Standard Error of the Mean). Data distribution was assessed using the Shapiro–Wilk normality test. For comparisons between two independent groups, an unpaired Student's t‐test was used for normally distributed data, whereas the Mann–Whitney U test was used for non‐normally distributed data. For paired comparisons, the Wilcoxon signed‐rank test was used when data were not normally distributed. For comparisons among three or more groups, one‐way or two‐way ANOVA followed by Tukey's multiple comparisons test was performed, as appropriate. Repeated‐measures ANOVA was used to compare changes in response variables over time. All statistical analyses were performed using GraphPad Prism software (GraphPad Software Inc.). A *p* value < 0.05 was considered statistically significant.

## 3 Results

### 3.1 Disruption of ER Homeostasis and Pak1 Activation in the Progression of HFpEF

**FIGURE 1:**
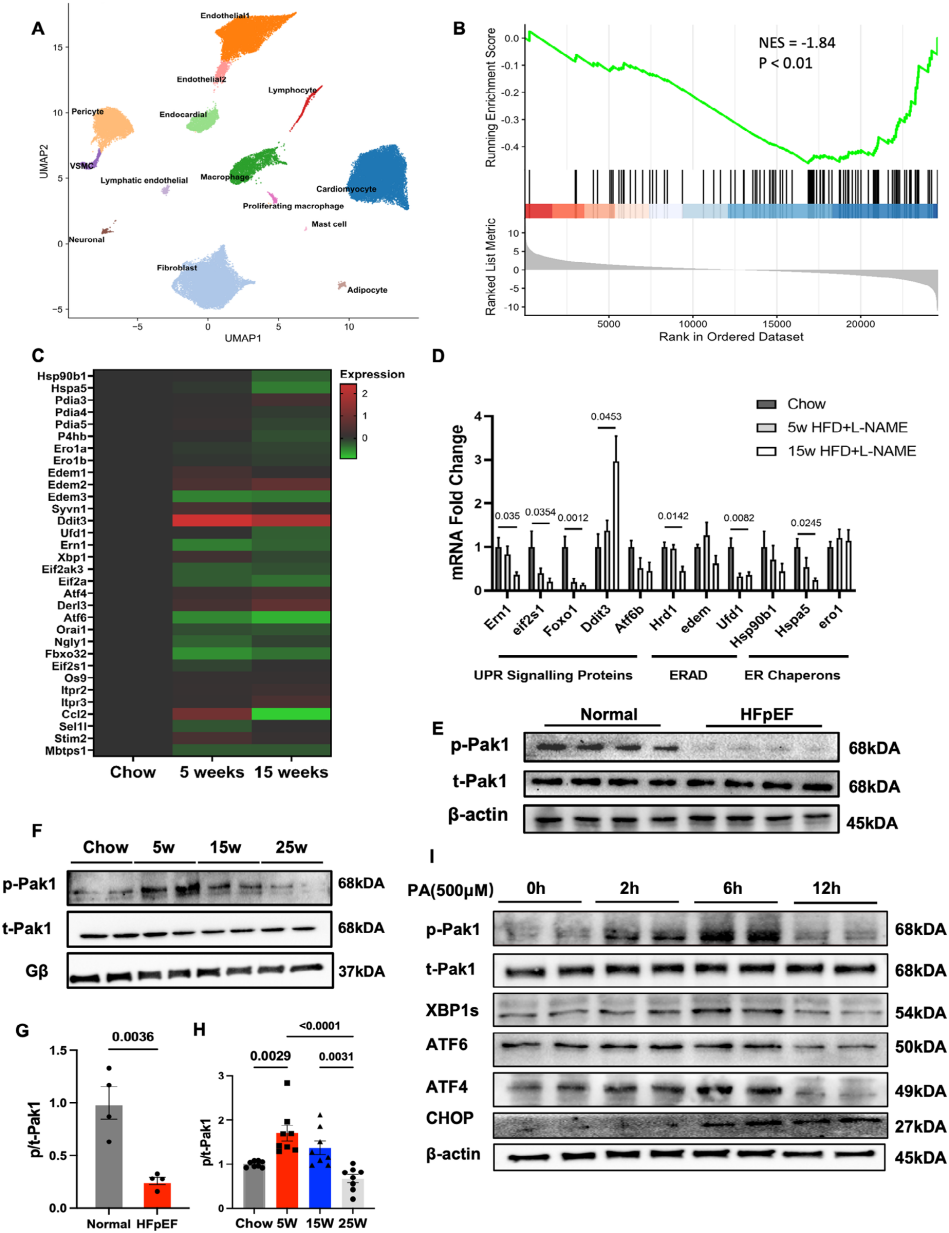
Pak1 and UPR related genes were downregulated during HFpEF progression. (A) UMAP of cardiomyocytes isolated from mouse hearts subjected to HFD+L‐NAME treatment, illustrating clustering based on transcriptomic profiles. (B) Gene set enrichment analysis (GSEA) of single‐nucleus RNA sequencing reveals significant downregulation of the “HALLMARK UNFOLDED PROTEIN RESPONSE” pathway in cardiomyocytes from the HFpEF patients compared to controls. (C) Heatmap of endoplasmic reticulum (ER)‐related gene expression in mouse hearts after 5 and 15 weeks of HFD + L‐NAME treatment, n = 3 mice per group. (D) qPCR results of mice heart tissues, n = 3‐5 per group. (E,G) Representative western blots and quantification of Pak1 from human heart samples. n = 4 samples per group. (F,H) Representative western blots and quantification of Pak1 from the mouse heart tissues. n = 5 mice per group. (I) Representative western blots of proteins from primary mouse cardiomyocytes. Data are presented as means ± SEM. Statistical analysis was by one‐way ANOVA followed by Tukey's multiple comparisons test.

To identify cellular pathways associated with HFpEF pathogenesis, we interrogated a human myocardial snRNA‐seq dataset generated from HFpEF patients and non‐failing donor controls [35]. Because cardiomyocytes are highly dependent on ER‐mediated protein quality control to maintain contractile function and cellular homeostasis, we examined ER‐related pathways specifically within the cardiomyocyte cluster. GSEA revealed a significant negative enrichment of the “HALLMARK UNFOLDED PROTEIN RESPONSE” pathway (NES = −1.84, p < 0.01), indicating a broad suppression of ER protein quality‐control machinery in HFpEF cardiomyocytes (Figure 1A,B). These findings suggest an impaired adaptive stress response within the myocardium, potentially contributing to the metabolic and structural remodeling observed in HFpEF. To further investigate, we analyzed ER‐related gene expression by qPCR at 5 and 15 weeks of HFD+L‐NAME treatment (Figure 1C,D). We found that by 15 weeks, the expression of ER‐related genes was reduced, accompanied by a significant upregulation of *Ddit3*, a gene encoding the pro‐apoptotic protein CHOP. Given the centrality of impaired ER function, we focused on potential upstream regulators. Pak family kinases are pivotal in stress signaling, and our prior work established that Pak2 modulates the IRE1‑XBP1s branch of the unfolded protein response [34]. However, the role of Pak1 in ER homeostasis remained unexplored. We first assessed Pak1 activation in human myocardium and found that phosphorylated Pak1 (p‑Pak1, Thr423) levels were significantly decreased in HFpEF patients compared to non‐failing controls (Figure 1E,G), establishing its clinical relevance. To delineate the temporal dynamics of Pak1 activation, we examined the HFD+L‐NAME‐induced HFpEF mouse model over a 25‐week period. Western blot analysis revealed that cardiac p‐Pak1 was significantly elevated after 5 weeks of HFD+L‐NAME treatment. However, p‐Pak1 levels progressively declined with prolonged exposure to metabolic stress, returning toward baseline at 15 weeks and becoming significantly reduced at 25 weeks (Figure 1F,H). These results suggest that early p‑Pak1 elevation may represent an adaptive response to metabolic‐hemodynamic stress, whereas its subsequent decline reflects a loss of compensatory capacity under prolonged stress.

Furthermore, palmitic acid (PA), a saturated free fatty acid (FFA) elevated in obesity, was administered to H9c2 cells to induce metabolic stress. The 3‐(4,5‐dimethylthiazol‐2‐yl)‐2,5‐diphenyltetrazolium bromide (MTT) assay showed that PA treatment at 125–500 µm reduced cell viability in a dose‐dependent manner (Figure S1A). Given that lipotoxic stress is closely linked to disruption of ER proteostasis, we next examined whether PA‐induced metabolic stress alters Pak1 activation and UPR signaling in primary mouse cardiomyocytes. Western blot analysis showed that PA induced dynamic activation of Pak1 and UPR‐related effectors, including ATF4, ATF6, and XBP1s, with early activation observed within 2 h and sustained up to 6 h before declining at later time points (Figure 1I; Figure S1B–F). In addition, the pro‐apoptotic protein CHOP was increased after 6 h of PA treatment (Figure 1I; Figure S1B–F). These findings suggest that Pak1 activation is dynamically associated with UPR regulation during lipotoxic metabolic stress, supporting a potential role for Pak1 in ER stress adaptation during HFpEF progression.

### 3.2 Pak1 Deficiency Accelerates HFpEF Development

**FIGURE 2:**
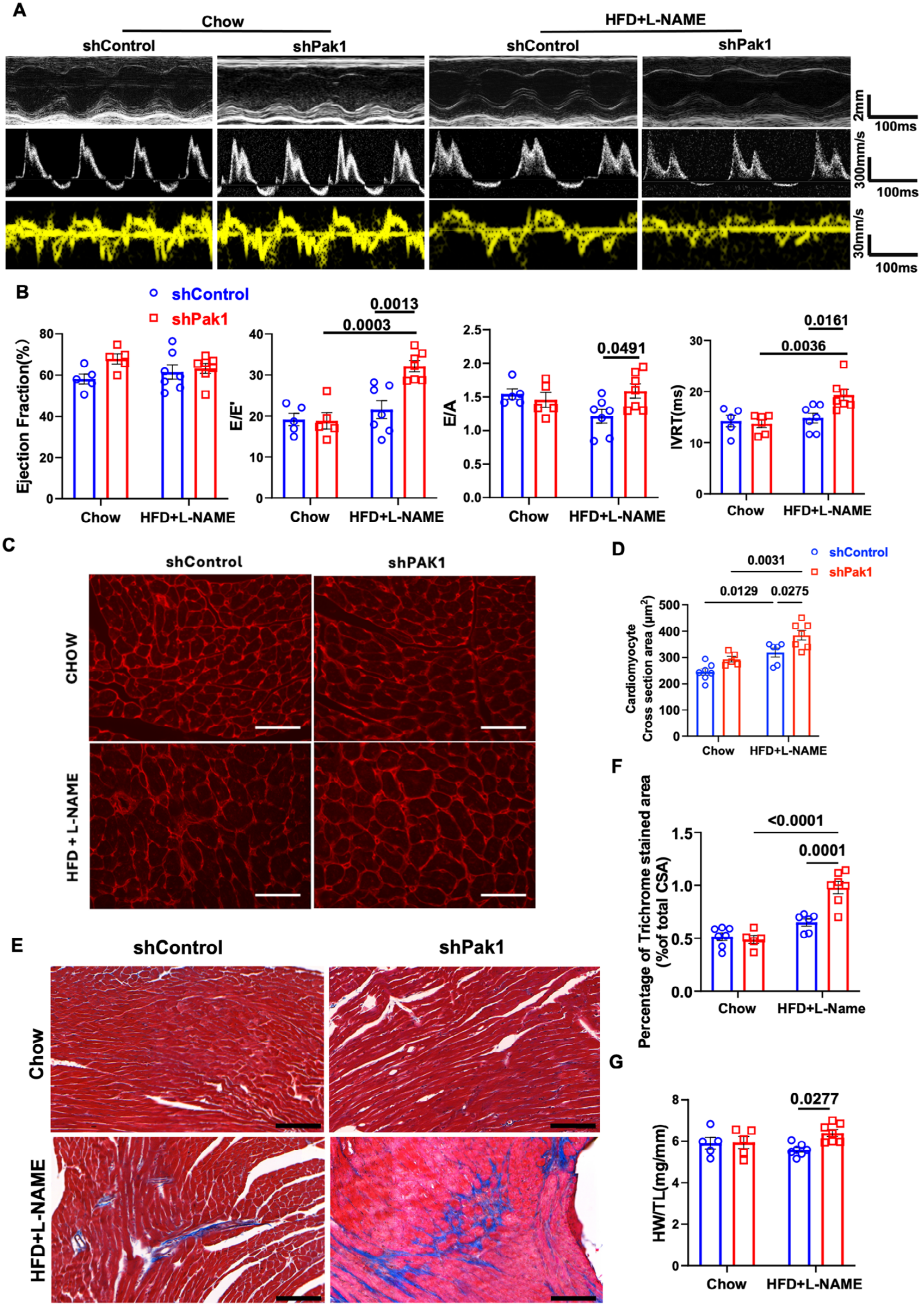
Knockdown of Pak1 in the heart impaired diastolic function at 6 weeks of HFpEF stress. (A) Representative image of echocardiography at 6 weeks. Top: M‐mode of short axis view, Middle: Doppler of E and A wave, Bottom: Tissue Doppler of E and e’ wave. (B) Preserved systolic function and reduced diastolic function in the left ventricle were detected by echocardiography at week 6. n = 5 to 7 mice per group. (C) Representative images of WGA staining. (D) The cross‐sectional areas of cardiomyocytes were assessed. (E) Representative images of Masson staining. (F) The percentage of fibrosis area was measured in transversal sections stained with Masson's trichrome. (G) The heart weight‐to‐tibia length ratio was calculated. Scale bars were set at 50 µm (WGA) and 100 µm (Masson). n = 5 to 8 samples per group. Data are presented as means ± SEM. Statistical analysis was by two‐way ANOVA followed by Tukey's multiple comparisons test.

For the functional examination of Pak1, we knocked down Pak1 in the cardiomyocytes of mice through AAV‐shPak1 injection. After 2 weeks, approximately 60% knockdown efficiency was achieved (Figure S2A–D). Both AAV9‐shPak1 and AAV9‐shControl mice exhibited a significant increase in body weight at 6 weeks of treatment with the HFD and L‐NAME (Figure S2E,F). Intraperitoneal glucose tolerance test (IGTT) showed that blood glucose levels were increased in both groups treated with HFD+L‐NAME, with shPak1 mice exhibiting a more pronounced trend (Figure S2G,H), indicating impaired glucose tolerance. Furthermore, systolic and diastolic blood pressure were significantly increased in both shControl and shPak1 mice treated with HFD+L‐NAME compared to mice fed with a chow diet (Figure S2I,J). There was no difference in blood pressure between the shControl HFD+L‐NAME and shPak1 HFD+L‐NAME group, indicating that deficiency of Pak1 does not influence the blood pressure. More importantly, a notable decrease in diastolic function was recorded in shPak1 mice compared with shControl mice after 6‐week treatment based on echocardiography parameters, including E/e’, E/A, and IVRT (Figure 2A,B). However, there were no differences of IVRT, E/e’ and E/A ratio observed between the shControl chow and shControl HFD+L‐NAME groups. No significant differences in heart rate were observed among the groups (Figure S2K). Throughout the 6‐week treatment period, ejection fraction remained within normal range for all groups (Figure 2B). These demonstrated that knockdown of Pak1 in the heart may accelerate the development of the HFpEF phenotype when combined with HFD+L‐NAME treatment. Previous studies have shown that HFpEF phenotype in wild‐type mice occurs at 15 weeks HFD+L‐NAME treatment [32], however, in Pak1 knockdown mice, there was an early diastolic dysfunction at 6 weeks of HFpEF treatment. Histology analysis was further performed to investigate the structural changes in Pak1 knockdown mice after HFpEF phenotype occurred at 6 weeks. WGA staining showed that the cross‐sectional area in the left ventricle was significantly increased in shPak1 HFD+L‐NAME compared to shPak1 chow and shControl HFD+L‐NAME group (Figure 2C,D). Additionally, the ratio of heart weight to tibia length further confirmed the occurrence of hypertrophy in Pak1 knockdown mice treated with HFD+L‐NAME when compared to the shControl HFD+L‐NAME group (Figure 2G). Furthermore, the extent of fibrosis in Pak1 knockdown mice was markedly greater compared to control mice after HFD+L‐NAME treatment (Figure 2E,F). These findings suggest that the Pak1 knockdown in cardiomyocytes could accelerate the progression of cardiac hypertrophy and fibrosis in response to HFD+L‐NAME treatment.

### 3.3 Pak1 Deficiency Leads to Pulmonary Injury

Pulmonary congestion is a hallmark clinical sign of inadequate control or decompensation in patients with HFpEF. We also examined the function of the lungs using ultrasound analysis. The heightened brightness and the presence of B‐lines were detected in Pak1 knockdown mice following 6 weeks of HFD+L‐NAME diet treatment (Figure S3A). Lung injury was quantified using a modified lung ultrasound score based on B‐lines, loss of A‐lines, increased lung echogenicity, and pleural line thickening, with a total score ranging from 0 to 7. The lung ultrasound score for the shPak1 HFD+L‐NAME group was significantly elevated compared to the control groups (Figure S3B). Additionally, the lung weight to tibia length ratio (LW/TL) was markedly higher in the shPak1 HFD+L‐NAME group than in the shPak1 chow group (Figure S3C). These findings indicate that pulmonary oedema developed in the Pak1 knockdown mice after 6 weeks of HFD+L‐NAME treatment. Histological analysis of pathological changes in lung tissues showed remarkedly thicken alveolar walls, fibrosis around the bronchi, and a disruption of the alveolar structure in shPak1 HFD+L‐NAME group (Figure S3D–G). These results show that the 6‐week HFD+L‐NAME challenge after Pak1 knockdown induced lung injury, which is commonly seen in HFpEF patients. The mechanisms underlying the lung injury are likely related to diastolic dysfunction, which increases left atrial pressure and leads to pulmonary hypertension. The inhibition of NO synthase by L‐NAME contributes to an increase in vascular tone and pulmonary blood pressure, resulting in the direct development of pulmonary hypertension and lung injury.

### 3.4 Deletion of Pak1 in Cardiomyocytes Leads to Impaired ER Function and Apoptosis

**FIGURE 3:**
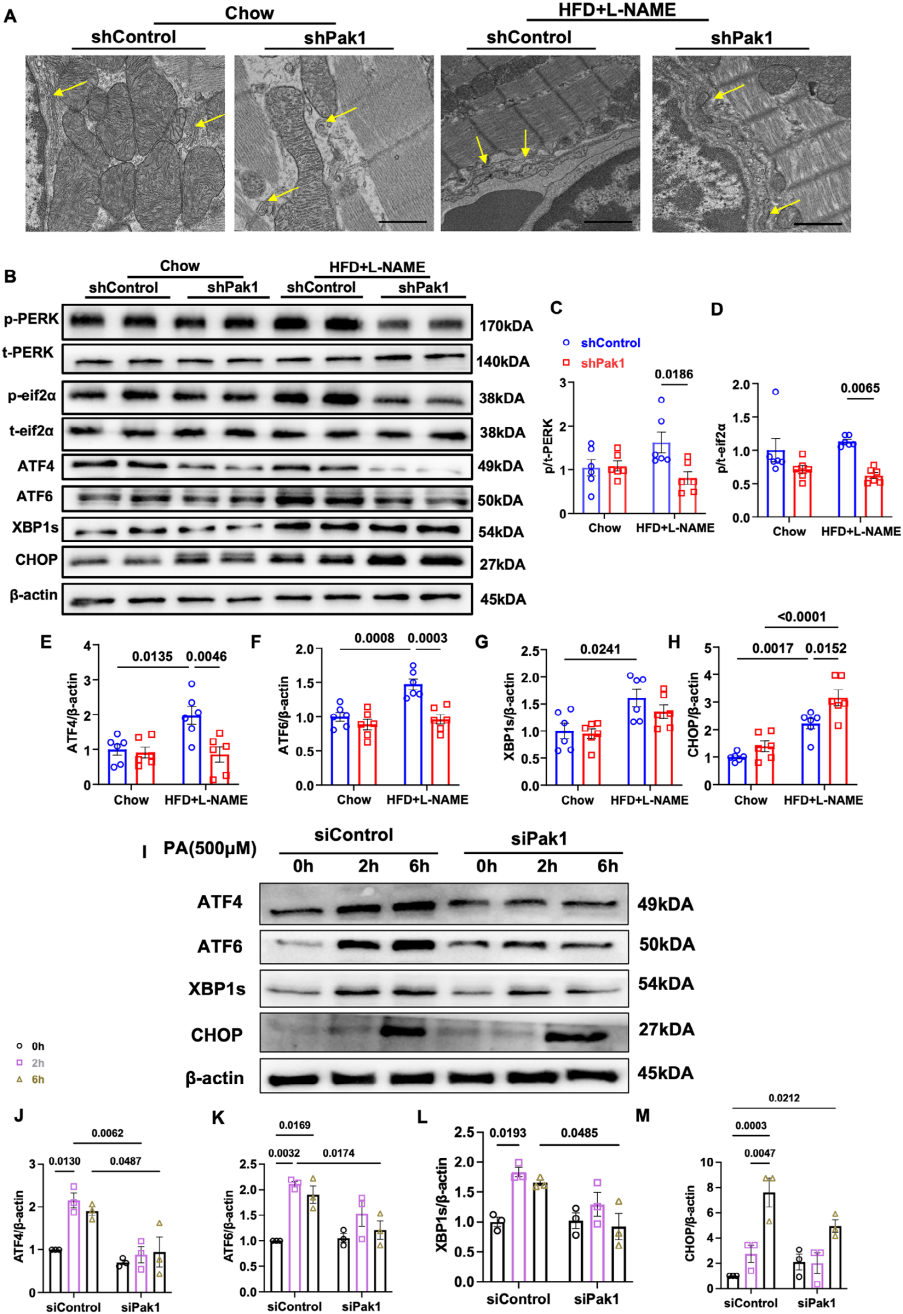
Pak1 knockdown impaired ER function and promoted cardiomyocyte apoptosis. (A) Electron microscopy showed expanded ER lumens of Pak1 knockdown mouse hearts after 6 weeks of HFD+L‐NAME treatment. Arrowheads show normal ER (arrows) and fragmented ER with vacuolization. Scale bars = 1 µm. (B) Representative western blots. (C‐H) Quantification of ER stress‐related proteins. n = 6 mouse samples per group. (I) Representative western blots of t‐Pak1 and UPR pathway effectors and (J‐M) Quantification of panel (I) in AC16 cells. n = 3 samples per group. Data are presented as means ± SEM. Statistical analysis was by two‐way ANOVA followed by Tukey's multiple comparisons test.

As a chronic disease, HFpEF is characterized by a sustained imbalance of cellular homeostasis. The ER, a critical organelle for maintaining intracellular homeostasis, is highly sensitive to various stress stimuli. Given that homeostatic imbalance often results from persistent cellular stress, we investigated the impact of shPak1 on ER morphology as a potential contributor to ER stress. To this end, we employed transmission electron microscopy (TEM) to assess ultrastructural changes in the ER following HFD+L‐NAME treatment in shPak1 mice. An increased proportion of dilated ER cisterns and partially cytoplasmic vacuolization were detected in shPak1 mice following 6‐week HFD+L‐NAME treatment (Figure 3A). The normally elongated and continuous structure of the ER appeared distorted after Pak1 knockdown during HFD+L‐NAME in the shControl group. We further evaluated UPR‐related protein expression in Pak1 knockdown mice (Figure 3B–H). The results showed that the three downstream effectors (ATF4, ATF6, XBP1s) of the UPR pathway were increased after 6 weeks of HFD+L‐NAME treatment compared with the chow group, suggesting activation of the UPR in the pre‐disease phase. Interestingly, following Pak1 knockdown, ATF4 and ATF6, but not XBP1s, were downregulated. This corresponds to the time point when alterations in cardiac diastolic function are observed. Since ATF4 is primarily regulated through the PERK–eIF2α branch of the UPR, we next examined whether the reduction in ATF4 was associated with impaired upstream PERK–eIF2α signaling. Consistently, phosphorylation levels of PERK and eIF2α were reduced in Pak1 knockdown hearts during HFpEF progression (Figure 3B–D), suggesting that Pak1 deficiency selectively compromises the PERK–eIF2α–ATF4 axis. This blunted adaptive UPR coincided with a more pronounced induction of CHOP, supporting maladaptive ER stress and increased apoptosis (Figure 3H). To investigate this further, we transfected siPak1 in AC16 cells, which were then exposed to PA for 0, 2, and 6 h. The expression of three UPR effectors observed in AC16 cells was consistent with that detected in cardiac tissue from the animal model (Figure 3I–M). These results suggest that the acceleration of the HFpEF phenotype in the heart due to the knockdown of Pak1 is likely mediated through the blunting of the ATF4 and ATF6 during the ER stress response.

### 3.5 Cardiac‐Specific Pak1 Overexpression Is Sufficient to Alleviate HFpEF Induced Cardiac Dysfunction

**FIGURE 4:**
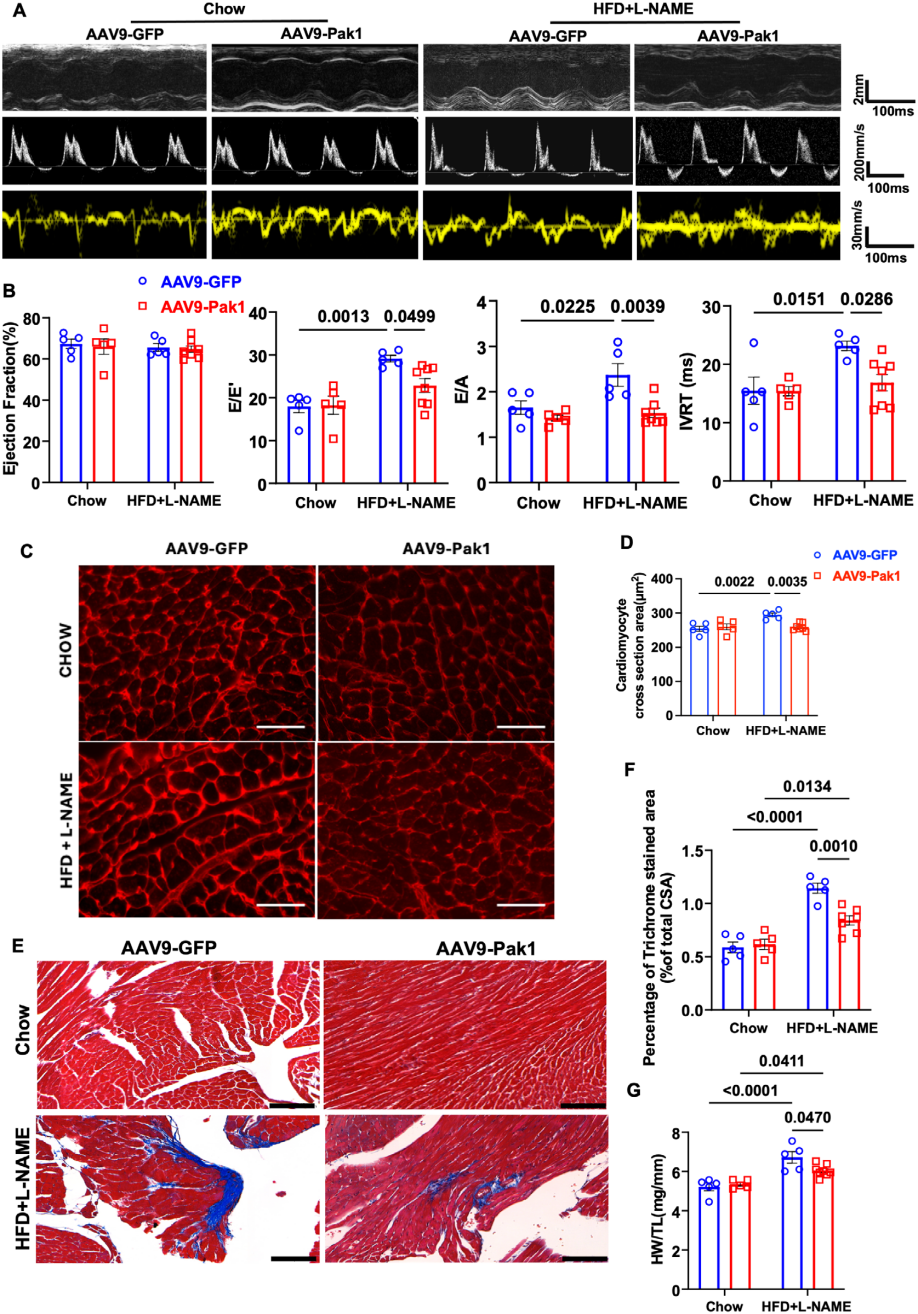
Pak1 overexpression preserved cardiac diastolic function following HFD+L‐NAME treatment. (A) Representative image of echocardiography at 15 weeks of HFpEF stress. Top: M‐mode of parasternal short axis view, Middle: Doppler image of E and A wave, Bottom: Tissue Doppler of E and e’ wave. (B) Systolic function and diastolic function were analyzed. n = 5 to 8 mice per group. (C) Section morphology of hearts stained with WGA and (D) quantitative analysis. (E) Cardiac fibrosis stained with Masson and (F) quantitative analysis. (G) Proportion of heart weight to tibia length. Scale bars were set at 50 µm (WGA) and 100 µm (Masson). n = 5 to 8 samples per group. Data are presented as means ± SEM. Statistical analysis was by two‐way ANOVA followed by Tukey's multiple comparisons test.

Given that loss of Pak1 in the heart induces HFpEF and is associated with ER stress, we next investigated whether Pak1 overexpression could mitigate these pathological effects. AAV‐Pak1 was injected into mice via the tail vein, and the western blot confirmed that Pak1 was overexpressed in the heart only (Figure S4A–C). Previous studies have demonstrated that 15 weeks of HFD+L‐NAME can induce an HFpEF phenotype [18]. In our study, mice were injected with AAV9‐Pak1 for two weeks following 15‐week HFD+L‐NAME treatment. Both body weight and glucose tolerance were impaired in AAV9‐GFP and AAV9‐Pak1 mice following HFD+L‐NAME treatment (Figure S4D–G). Blood pressure was also increased after HFD+L‐NAME treatment in both AAV9‐GFP and AAV9‐Pak1 mice (Figure S4H,I). The heart function was further assessed using echocardiography throughout the 15‐week treatment. The echocardiography assessment revealed that systolic function remained normal across all four groups throughout the duration of the study (Figure 4A,B). After 15 weeks, diastolic function remained normal in the AAV9‐Pak1 group with chow diet. However, the AAV9‐GFP group experienced a significant decline following HFD+L‐NAME, as indicated by increased E/e’, E/A and IVRT (Figure 4A,B), while diastolic function was preserved in Pak1 overexpression mice. This suggests that Pak1 overexpression has a protective role in preserving diastolic function against the detrimental effects of HFD+L‐NAME.

Next, histological analysis was conducted to further explore the impact of Pak1 on inhibiting cardiac hypertrophy and fibrosis. WGA and Masson staining showed that both the AAV9‐GFP and AAV9‐Pak1 groups showed no significant differences under chow diet conditions (Figure 4C–F). However, after HFD+L‐NAME treatment, the cross‐sectional area and myocardial fibrosis of the AAV9‐GFP mice significantly increased compared to the AAV9‐GFP chow group (Figure 4D,F), but Pak1 overexpression evidently ameliorated myocardial fibrosis and myocardial hypertrophy. In terms of HW/TL ratio, no differences were found between the AAV9‐GFP and AAV9‐Pak1 groups under chow diet conditions. After 15 weeks of HFD + L‐NAME treatment, the HW/TL ratio was significantly increased in control mice (Figure 4G). In contrast, mice with Pak1 overexpression did not show this increase. Collectively, these findings suggest that Pak1 overexpression mitigates cardiac fibrosis and hypertrophy induced by HFD+ L‐NAME challenge.

### 3.6 Pak1 Overexpression Alleviated Lung Injury during HFpEF

H&E and Masson staining were used to investigate the effect of Pak1 on lung injury. Alveolar septal thickening and a small amount of collagen fiber organization were observed in the lung interstitium in AAV9‐GFP under 15 weeks of HFD+L‐NAME challenge compared with thechow group (Figure S5A–D). However, these pathologic changes were fewer in that of AAV9‐Pak1 group. The LW/TL ratio is significantly elevated in HFD+L‐NAME‐treated AAV9‐GFP mice compared to chow, indicating lung congestion (Figure S5E). Overexpression of Pak1 partially reduces this ratio under HFD+L‐NAME conditions. The results supported that overexpression of Pak1 appears to mitigate lung injury, suggesting a protective role in preventing pulmonary structural remodeling under HFD+L‐NAME challenge.

### 3.7 Pak1 Overexpression Improved the ER Following 15 Weeks of HFD+L‐NAME Treatment

**FIGURE 5:**
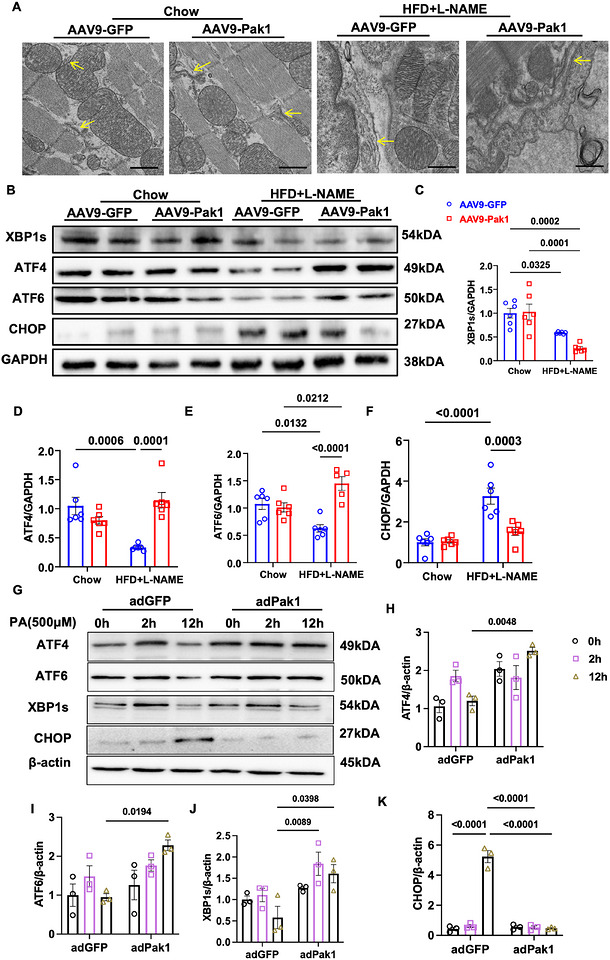
Pak1 overexpression potentiated protective UPR through ATF4 and ATF6 signaling. (A) Representative TEM images from n = 3 samples per group. Yellow arrows indicate ER. Scale bar = 1 µm. (B) Representative western blot of ER stress‐related proteins. (C–F) Quantification of ER stress‐related proteins. n = 6 samples per group. (G) Representative western blots of t‐Pak1 and UPR pathway effectors, and (H–K) Quantification of panel (G). n = 3 samples per group. Data are presented as means ± SEM. Statistical analysis was by two‐way ANOVA followed by Tukey's multiple comparisons test.

Previous studies indicate that ER stress resulting from the accumulation of misfolded proteins plays a significant role in the onset and progression of HFpE [36]. Therefore, transmission electron microscopy was used to examine ER morphology. As expected, we found ultrastructural changes with ER stress features such as swollen ER with remarkably enlarged lumen of HFpEF mice, this morphology was improved after Pak1 overexpression (Figure 5A). There was no significant difference in the UPR pathway effectors (XBP1s, ATF4, ATF6) between AAV9‐GFP and AAV9‐Pak1 under basal level (Figure 5B–E). However, these proteins were decreased after 15 weeks HFD+L‐NAME treatment in AAV9‐GFP group compared with the chow group. Pak1 overexpression in the heart prevented the reduction in ATF4 and ATF6, but not in XBP1s (Figure 5B–E). Finally, CHOP expression, a key marker of ER stress‐induced apoptosis, was significantly increased in HFD+L‐NAME AAV9‐GFP mice, and this increase was partially mitigated by Pak1 overexpression (Figure 5F). These findings suggest that while HFD+L‐NAME treatment reduces certain markers of ER stress (XBP1s, ATF4, and ATF6), it paradoxically increases CHOP expression, indicating enhanced ER stress‐induced apoptosis. Overexpression of Pak1 prompts the UPR by partially restoring the expression of ATF4, ATF6, and mitigating apoptosis, highlighting a protective role of Pak1 in heart tissues under HFD+L‐NAME conditions. To assess the functional impact of Pak1 overexpression on the UPR under metabolic stress, AC16 cells overexpressing Pak1 via viral transduction (Ad‐Pak1) were treated with PA for 0, 2, and 6 h. Western blot analysis revealed the activation of UPR markers ATF4, ATF6, and XBP1s, as well as the apoptotic marker CHOP. Compared to cells transduced with ad‐GFP, ad‐Pak1 significantly enhanced the expression of ATF4 and ATF6 in a time‐dependent manner following PA treatment (Figure 5G–J). Notably, CHOP expression, which reflects ER stress‐induced apoptosis, was markedly reduced in ad‐Pak1 cells, suggesting a protective role of Pak1 overexpression (Figure 5K). These findings suggest that Pak1 plays a critical role in maintaining ER homeostasis and mitigating apoptotic responses during metabolic stress, providing potential therapeutic insights for conditions associated with ER stress.

### 3.8 Pak1 Regulates Adaptive ISR Signaling via the ERK1/2‐MNK1‐eIF4E Axis

**FIGURE 6:**
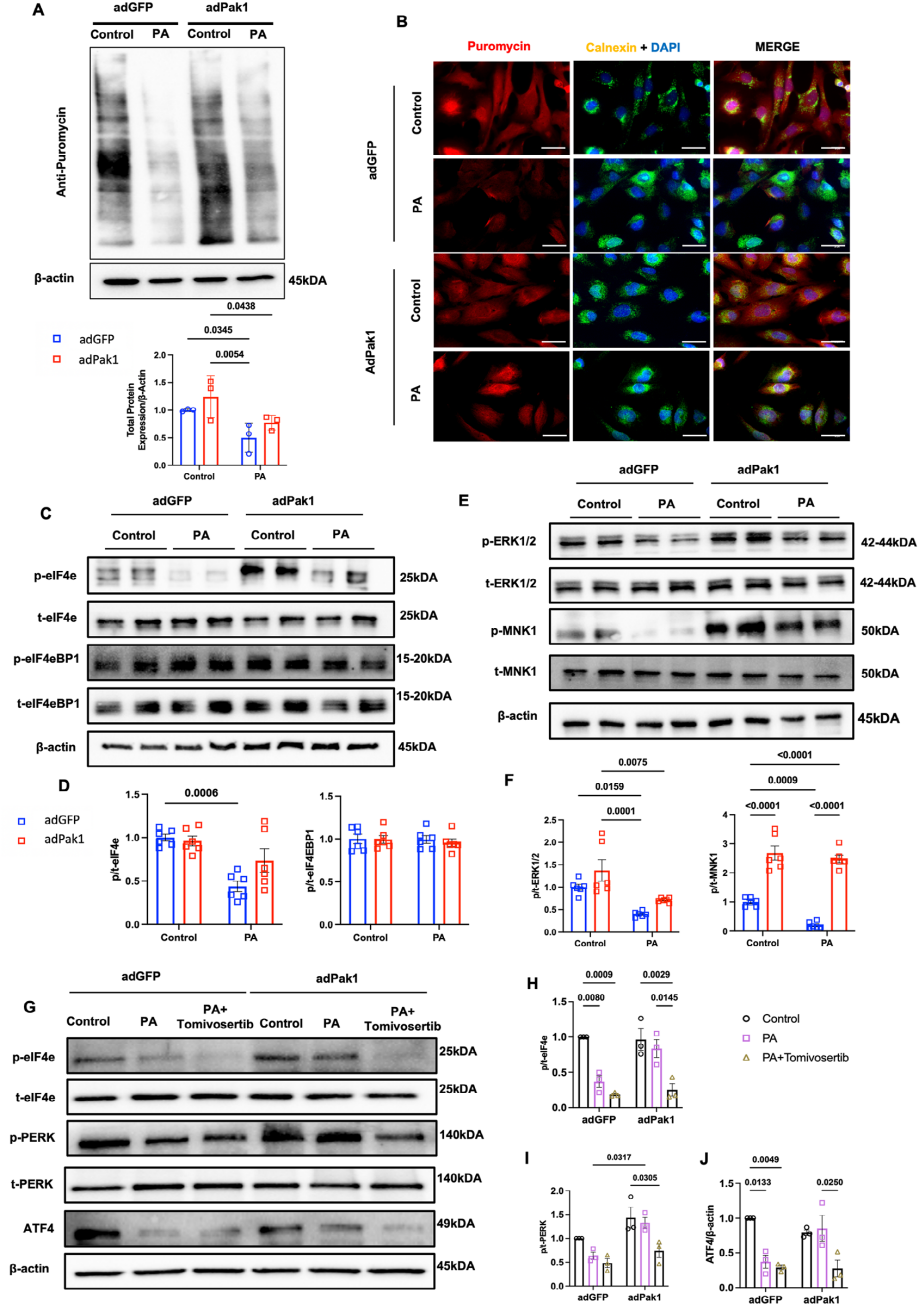
Pak1 overexpression restores protein synthesis via ERK‐MNK1‐eIF4E signaling in PA‐treated AC16 cells. (A) Representative puromycin incorporation assay and corresponding quantification of total protein synthesis in AC16 cells. n = 3 samples per group. (B) Immunofluorescence images showing puromycin for nascent peptide (red) and Calnexin for ER (green). Nuclei were counterstained with DAPI (blue). Scale bar = 20 µm. (C,D) Western blots and quantification of the total and phosphorylation of eIF4E, and eIF4EBP1 expression. n = 6 samples per group. (E,F) Western blots and quantification of MNK1 and ERK1/2. n = 6 samples per group. (G–J) Western blots and quantification of phosphorylated eIF4E, phosphorylated PERK, and ATF4 expression in adGFP‐ and adPak1‐transduced cells treated with PA in the presence or absence of the MNK1/2 inhibitor tomivosertib. n = 3 samples per group. Data are presented as means ± SEM. Statistical analysis was by two‐way ANOVA followed by Tukey's multiple comparisons test.

Given that Pak1 deficiency selectively impaired PERK‐eIF2α‐ATF4 signaling, and that Pak1 overexpression enhanced eIF2α phosphorylation under PA‐induced metabolic stress (Figure S6A,B), we next investigated whether Pak1 influences the integrated stress response (ISR), a key adaptive programme downstream of PERK that coordinates translational control during ER stress. Because translational reprogramming is a hallmark of ISR activation, we first assessed global protein synthesis using puromycin incorporation assays. Under basal conditions, Pak1 overexpression did not significantly alter puromycin incorporation compared with control cells (Figure 6A). PA treatment for 12 h reduced overall protein synthesis in both control and Pak1‐overexpressing cells; however, Pak1‐overexpressing cells retained relatively higher puromycin incorporation than PA‐treated control cells, suggesting that Pak1 helps preserve translational activity under metabolic stress. Immunofluorescence analysis demonstrated substantial overlap between puromycin incorporation and ER staining, indicating that a considerable proportion of newly synthesized proteins were associated with the ER compartment (Figure 6B). Pak1 overexpression partially preserved this ER‐associated nascent peptide signal following PA treatment for 12 h, suggesting maintenance of translational activity within ER‐rich regions during metabolic stress. To explore the molecular mechanisms underlying this translational regulation, we examined key regulators of cap‐dependent protein synthesis. While phosphorylation of eIF4E‐BP1 was not significantly altered by Pak1 overexpression, phosphorylation of eIF4E was markedly increased, suggesting that Pak1 may regulate translation through an eIF4E‐dependent mechanism (Figure 6C,D). Because eIF4E phosphorylation is primarily mediated by the ERK1/2‐MNK1 signaling axis, we subsequently examined components of this pathway. Pak1 overexpression increased phosphorylation of ERK1/2 and eIF4E under both basal and PA‐treated conditions (Figure 6E,F), while MNK1 phosphorylation showed a similar pattern, supporting activation of the ERK1/2‐MNK1‐eIF4E translational pathway. We next investigated whether this translational signaling axis contributes to the activation of adaptive ISR signaling downstream of Pak1. To test this, Pak1‐overexpressing cardiomyocytes were treated with the MNK1/2 inhibitor tomivosertib. MNK1 inhibition attenuated Pak1‐induced PERK phosphorylation and reduced ATF4 expression, suggesting that Pak1‐mediated activation of adaptive PERK‐ATF4 signaling is dependent on MNK1 activity (Figure 6G–J). Together, these findings indicate that Pak1 enhances translational activity through ERK1/2–MNK1–eIF4E signaling while simultaneously activating a PERK‐dependent adaptive ISR programme characterized by increased ATF4 expression. This coordinated response may allow cardiomyocytes to maintain selective protein synthesis and proteostatic adaptation during metabolic stress.

### 3.9 Therapeutic Targeting of Pak1 Attenuates Cardiac Dysfunction and Promotes UPR in HFpEF Mouse Model

**FIGURE 7:**
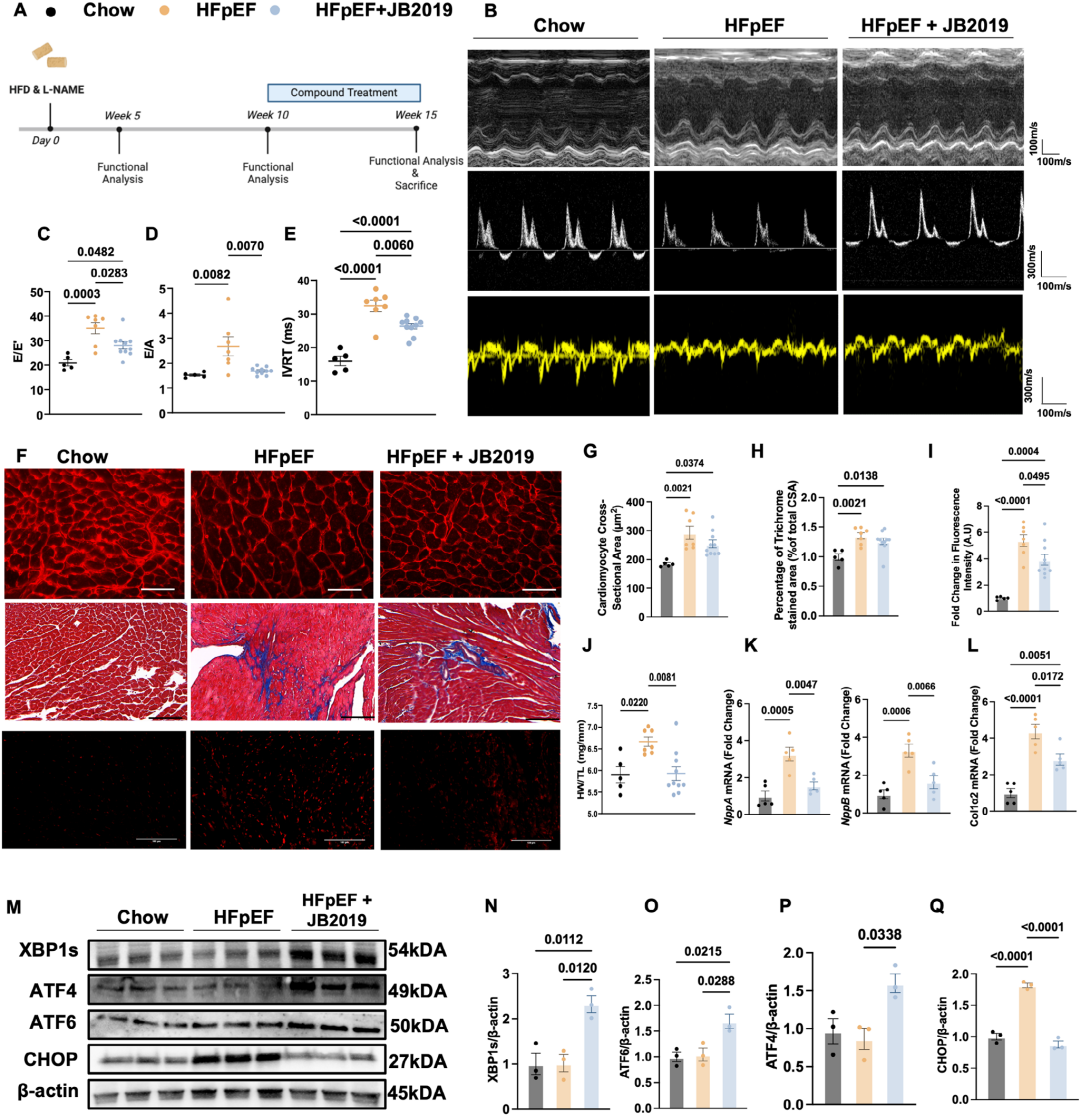
Therapeutic targeting of Pak1 with compound JB2019 improved cardiac function and alleviated ER stress in HFpEF. (A) Experimental Overview. After 10‐weeks of HFpEF treatment, mice were subjected to compound treatment until 15‐weeks. Functional analysis was performed every 5 weeks. (B) Representative left ventricular M‐mode (top), pulse wave doppler (middle), and tissue Doppler (bottom) electrocardiographic films. (C–E) Tissue doppler analysis of E/e’ wave, E/A wave, and IVRT at 15 weeks. n = 5–10 mice per group. (F) Representative images of WGA staining (Scale bar = 50 µm), Masson's trichrome staining (Scale bar = 100 µm), and DHE staining (Scale bar = 100 µm). (G) Quantification of WGA staining to assess cardiomyocyte cross sectional area. (H) Quantification of Masson's trichrome staining to assess interstitial fibrosis. (I) Quantification of DHE staining to assess superoxide generation. (J) Heart weight/tibial length ratio. n = 5‐10 samples per group. (K–L) Relative mRNA expression of hypertrophic (NppA and NppB) and fibrotic (Col1a2) gene markers. n = 5 samples per group (M) Representative immunoblots of key markers of the ER stress response pathway and (N‐Q) Quantification of panel (M). n = 3 samples per group. Data are presented as means ± SEM. Statistical analysis was by two‐way ANOVA followed by Tukey's multiple comparisons test.

To test the therapeutic potential of our findings, we treated mice with a recently developed Pak1‐activating compound JB2019. After 10 weeks of dietary intervention, mice were subjected to compound treatment until 15 weeks (Figure 7A). Echocardiography revealed improved cardiac diastolic function compared with the HFpEF group (Figure 7B–E). Examination of the ratio of heart weight to tibia length and histology analyses demonstrated that JB2019 treatment attenuated HFpEF‐induced cardiomyocyte hypertrophy and cardiac fibrosis (Figure 7F–J). The cardiac tissue displayed the anticipated changes in expression of fetal gene program markers of hypertrophy (Figure 7K,L). Dihydroethidium (DHE) staining results indicated that JB2019 also reduced ROS production, a key trigger for cardiomyocyte apoptosis (Figure 7F,I). We also examined whether JB2019 could regulate the UPR pathway through mimicking Pak1 overexpression in the heart. Western blot results showed that JB2019 reversed the downregulation of UPR‐related proteins induced by HFpEF (Figure 7M–Q).

### 3.10 JB2019 Restores ER Function and Electrophysiology in Palmitic Acid–Treated Cardiac Organoids

**FIGURE 8:**
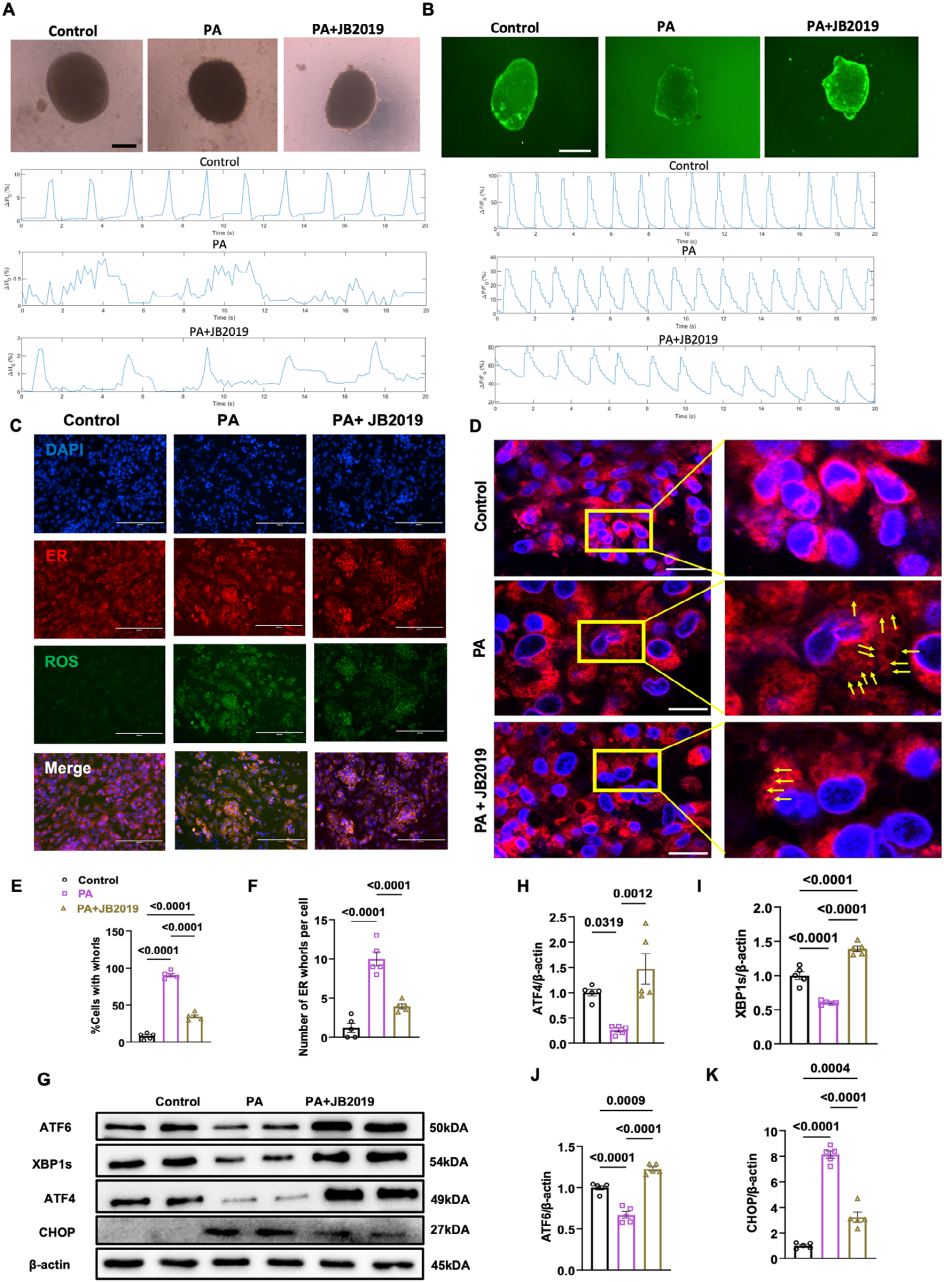
JB2019 alleviated PA‐induced ER stress in cardiac organoids. (A) Representative brightfield images of cardiac organoids beating activity, I indicates intensity. Scale bar = 1000 µm. (B) Representative calcium images and the synchronized contraction was shown by calcium flux frequency. Results were expressed as F/F_0_ (F_0_ refers to baseline intensity). Scale bar = 1000 µm. (C) Immunofluorescence images of hESC‐CMs with PA and JB2019 treatment showing colocalization of ROS (CellROX, green) and ER marker (ER Tracker, red); nuclear marker DAPI (blue). Scale bar = 200 µm. (D) Cardiac organoids stained with ER‐Tracker Red and DAPI blue visualized by confocal microscopy. Scale bar = 50 µm. (E) Cells from (D) were quantified for the percentage of ER whorls (n = 5 independent samples per group; >100 cells analyzed per sample). (F) Quantification of ER whorl number in cells from (D). n = 5 independent samples per group; >100 cells analyzed per sample. (G) Representative western blots of UPR pathway effectors and (H‐K) Quantification of panel (G). n = 5 independent samples per group; Data are presented as means ± SEM. Statistical analysis was by two‐way ANOVA followed by Tukey's multiple comparison test.

Rodents are commonly used in cardiomyopathy research, but significant species differences limit their relevance to human heart disease and drug safety assessment [37]. Human cardiac organoids offer a more accurate model, better mimicking tissue physiology with mature contractility, electrophysiology, and sarcomere structure [38, 39, 40]. We developed a cardiac organoid platform composed of hESC‐derived cardiomyocytes, cardiac fibroblasts, and endothelial cells at a defined 7:1:2 ratio, allowing the cells to self‐assemble into structures that closely resemble native human heart tissue (Figure S7A–E). We first examined the UPR activation after PA treatment at different time points (Figure S8A–F). Potent dose‐responsive Pak1 activation was then observed with more than double the expression in cardiac organoids after JB2019 treatment (Figure S8G,H). Next, we sought to characterize the functionality of organoids under PA conditions with JB2019 through electrophysiology for key markers. We first assessed the beating activity of organoids under bright‐field (Video 8A, Figure 8A), then the calcium transient activity of individual cardiomyocytes within organoids at day 10 of exposure to treatments with membrane‐permeable dye Fluo‐4 (Video 8B, Figure 8B). Organoids in all conditions exhibited substantial beating activities in both bright field and Fluo‐4 dye staining (Figure 8A,B). We found that, compared with the control group, PA led to asynchronous contraction and calcium transient recordings. There are altered rhythms, amplitude, and action potential observed under PA treatment. In comparison to the control group, PA treatment group displayed slower beating rates with decreased amplitudes (Figure 8A,B). This effect of PA is rescued by JB2019 co‐exposure, showing regular beating rates comparable to the control group and higher amplitudes than PA‐treated cardiac organoids. Since the ER is a crucial Ca^2+^ storage organelle, ER stress indeed led to calcium homeostasis imbalance in our experimental setting. PA treatment also exhibited increased cellular ROS predominantly accumulated in the ER, suggesting potential ER dysfunction (Figure 8C). We also observed that PA treatment induced ER whorls in nearly all cells within cardiac organoids, with an average of approximately 10 ER whorls per cell. Following JB2019 treatment, the proportion of cells exhibiting ER whorls was reduced to 35% (Figure 8D–F). Next, we found treatment of cardiac organoids with JB2019 (50 µm, 10 days) significantly reversed PA‐induced UPR down‐regulation, indicated by a significantly increased ATF4, XBP1s, and ATF6 together with markedly decreased CHOP expression (Figure 8G–K). Previous studies showed that prolonged ER stress induces formation of ER whorls, and this process is reversible upon removal of the ER stressor [41]. Collectively, JB2019 treatment has been shown to alleviate PA‐induced cardiac organoid dysfunction through regulating ATF4, XBP1s, and ATF6.

## 4 Discussion

HFpEF has emerged as the most prevalent type of heart failure, mainly driven by aging, obesity, hypertension, and metabolic comorbidities [42]. Despite its rising prevalence and substantial impact on cardiovascular health, HFpEF remains a major unmet clinical need, with limited effective treatments available. A deeper understanding of its pathogenesis is crucial to identify novel therapeutic targets. In this study, we identified Pak1 as a critical regulator of cardiac stress adaptation defending HFpEF progression. Using complementary in vivo, in vitro, and human cardiac organoid models, we demonstrate that Pak1 deficiency accelerates HFpEF development, whereas Pak1 overexpression or pharmacological activation preserves cardiac function and attenuates pathological remodeling. Mechanistically, our findings suggest that Pak1 promotes adaptive stress signaling through the ERK1/2‐MNK1‐eIF4E axis, which supports PERK‐ATF4‐dependent ISR activation and maintenance of ER proteostasis under metabolic stress conditions. Together, this study identifies Pak1 as a previously unrecognized regulator of adaptive ISR signaling and highlights Pak1 activation as a potential therapeutic strategy for HFpEF.

An intriguing finding of the present study is that Pak1 and Pak2 appear to regulate distinct ER stress response in the heart. In our previous work, we demonstrated that Pak2 protects the heart against ER stress predominantly through the IRE1‐XBP1s branch of the UPR by suppressing PP2A‐mediated dephosphorylation of IRE1, thereby maintaining IRE1 activity and adaptive ER signaling [43]. In contrast, the current study suggests that Pak1 preferentially regulates the PERK‐ATF4 arm of the stress response through a fundamentally different mechanism. Rather than directly modulating PERK activity, Pak1 activates the ERK1/2‐MNK1‐eIF4E translational pathway, leading to enhanced translational activity and subsequent activation of adaptive PERK‐ATF4 signaling. Mechanistically, Pak1 may engage the ERK1/2 cascade at the level of Raf and MEK. Earlier studies have shown that Pak1 can phosphorylate upstream components of the MAPK pathway, including c‐Raf at Ser338 and MEK1 at Ser298, thereby facilitating subsequent MEK1/2 and ERK1/2 activation [44]. In addition to this kinase‐dependent mechanism, Pak1 has also been reported to promote ERK1/2 activation in a kinase‐independent manner [45]. Overexpression of kinase‐dead Pak1 was sufficient to increase MEK1/2 and ERK1/2 phosphorylation, while Rac1 activation promoted formation of a Rac1–Pak1–MEK1 complex, suggesting that Pak1 can function as a scaffold to bring MEK into proximity with c‐Raf [45]. These findings indicate that Pak1 could couple upstream Rac/Cdc42 signaling to the Raf–MEK–ERK module through both catalytic and scaffolding mechanisms.

The distinct functions of Pak1 and Pak2 may reflect differences in both subcellular localization and substrate selectivity. Previous studies have shown that Pak1 is predominantly localized in the cytosol, whereas Pak2 is expressed in close proximity to the ER membrane [43]. Such compartmentalization may place Pak2 in a favorable position to directly regulate ER‐resident signaling complexes such as the PP2A‐IRE1 axis. In contrast, Pak1 appears to exert its effects through cytosolic translational regulatory networks involving ERK1/2‐MNK1‐eIF4E pathway. Importantly, activation of this pathway was associated with increased PERK phosphorylation and ATF4 expression, as evidenced by inhibition of MNK1 attenuating Pak1‐mediated PERK activation and ATF4 expression under stress state, supporting a functional link between translational control and adaptive ISR activation. The PERK–eIF2α–ATF4 arm of the ISR initially functions as a translational checkpoint that reduces global protein synthesis while selectively promoting stress‐adaptive transcriptional programmes involved in proteostasis, amino acid metabolism, redox homeostasis, and autophagy [46, 47, 48]. In cardiomyocytes, such signaling may be beneficial under pathological stress, as PERK has been reported to protect the heart against pressure overload‐induced heart failure, and cardiomyocyte‐specific loss of ATF4 exacerbates pressure overload‐induced cardiac dysfunction, fibrosis, and apoptosis, partly through impaired NADPH‐generating metabolic pathways and redox control [49, 50]. By contrast, sustained activation of the ATF4–CHOP axis is generally associated with unresolved ER stress and transition toward a terminal, pro‐apoptotic UPR [51]. CHOP promotes cell death through multiple mechanisms, including restoration of protein translation via GADD34, induction of oxidative stress‐related mediators such as ERO1α, regulation of pro‐apoptotic genes, and suppression of anti‐apoptotic Bcl‐2 signaling [52]. Therefore, the induction of PERK phosphorylation and ATF4 expression in the absence of substantial CHOP activation suggests that Pak1 engages a restrained ISR programme that supports cardiomyocyte adaptation rather than triggering maladaptive ER stress‐induced apoptosis. We therefore propose that Pak1 promotes a selective adaptive ISR characterized by coordinated enhancement of translational capacity and PERK‐ATF4 signaling, whereas Pak2 primarily supports ER proteostasis through direct regulation of the IRE1‐XBP1s pathway. This mechanism would be beneficial in HFpEF, where chronic metabolic stress imposes a sustained burden on protein quality‐control systems.

Our findings support a mechanism in which Pak1‐dependent activation of the ERK1/2–MNK1–eIF4E axis promotes selective translational reprogramming rather than a generalized increase in protein synthesis. Phosphorylation of eIF4E by MNK1 has been linked to stimulus‐dependent translation of specific subsets of mRNAs, particularly those involved in cellular adaptation, survival, and stress responses [53]. In cardiomyocytes, such a shift in translational output may increase the synthesis of selected ER‐targeted, membrane‐associated, or proteostasis‐related proteins, thereby transiently increasing protein‐folding demand within the ER. Rather than causing overt ER dysfunction, this moderate increase in ER protein flux may be sufficient to engage PERK as an adaptive sensor of proteostatic load. Activation of PERK would then phosphorylate eIF2α, attenuating bulk translation while permitting preferential translation of ISR‐responsive transcripts such as ATF4 through uORF‐dependent mechanisms [54]. In this model, Pak1‐driven eIF4E phosphorylation and PERK–eIF2α activation are not contradictory events, but represent two coordinated layers of translational control: the former reshapes selective mRNA translation, whereas the latter limits global protein synthesis and induces ATF4‐dependent adaptive programmes. This regulated ISR programme may enable cardiomyocytes to accommodate increased proteostatic and metabolic demand while avoiding CHOP‐dependent apoptotic signaling.

In this study, we first confirmed that PA, a well‐characterized saturated fatty acid, induces ER stress in cardiac organoids composed of human embryonic stem cell‐derived cardiomyocytes, endothelial cells, and fibroblasts. ER stress was evidenced by impaired ER structural integrity, disrupted calcium flux, and reduced beating activity. Treatment with a recently developed Pak activator JB2019, effectively ameliorated these defects, restoring functional and structural parameters. Together with our preceding mouse data, these findings provide compelling evidence for JB2019 therapeutic potential against PA‐induced ER stress in a human‐relevant 3D cardiac organoid model, underscoring its translational relevance to clinical application. The multicellular composition of cardiac organoids provides a supportive extracellular matrix and vascular‐like lumens, thereby more closely recapitulating the composition and physiology of the human heart compared to conventional 2D monolayer cultures. To further enhance the maturation and in vivo‐like microenvironment of this platform for disease modeling and drug testing, future work will incorporate additional cell types, such as immune and stromal cells, and employ hydrogel‐based culture systems. Overall, our results support the broad applicability of 3D cardiac organoids for modeling cardiac diseases, evaluating therapeutic candidates, and advancing personalized medicine approaches in combination with patient‐derived induced pluripotent stem cell technologies.

The promising effects of the novel Pak activator JB2019 in both the HFpEF model and PA‐treated COs underscore its therapeutic potential in modulating cardiac ER stress responses. The dual activation of Pak1 and Pak2 could be advantageous, considering the complementary yet distinct roles of Pak1 and Pak2 in coordinating ER stress pathways. However, given the overlapping but specialized functions of these two isoforms, development of isoform‐specific activators could improve therapeutic precision by enabling selective modulation of adaptive ER stress responses. Our findings, therefore, support the Pak family as central regulators of cardiac homeostasis in HFpEF. Moving forward, isoform‐selective Pak activators represent an important direction to optimize the efficacy and safety of Pak‐targeted therapies in heart failure and related conditions.

Several aspects of this work warrant further investigation. In the present study, cardiomyocyte‐directed Pak1 loss‐of‐function was achieved using AAV9‐mediated knockdown. This approach enabled efficient modulation of Pak1 expression in adult hearts and allowed assessment of its functional role during HFD+L‐NAME‐induced HFpEF progression, while avoiding the extended breeding and additional animal use required for generating new transgenic lines. Future studies using cardiomyocyte‐specific Pak1 conditional knockout models may provide complementary genetic validation and further refine the cell‐autonomous contribution of Pak1 to HFpEF pathogenesis. The present in vivo experiments were performed in male mice to maintain consistency across the experimental cohorts. Given the higher prevalence of HFpEF in females, particularly in older and post‐menopausal populations, it will be important in future studies to determine whether Pak1‐mediated regulation of ER/ISR signaling and cardiac remodeling is similarly operative in female HFpEF models. Such studies may also help define whether sex hormone‐dependent pathways interact with Pak1‐mediated stress adaptation. In addition, although this study primarily focused on ER stress and adaptive ISR signaling, ultrastructural analyses also revealed mitochondrial morphological alterations under HFpEF conditions. Considering the close functional interaction between the ER and mitochondria in regulating calcium handling, oxidative stress, and apoptosis, future work exploring whether Pak1 influences ER‐mitochondrial communication may provide further mechanistic insight into its cardioprotective effects.

## 5 Conclusions

This study identifies Pak1 as a key regulator of cardiac adaptation to metabolic stress in HFpEF. Mechanistically, Pak1 promotes adaptive ISR signaling through the ERK1/2‐MNK1‐eIF4E axis, leading to activation of the PERK‐ATF4 pathway and preserving ER proteostasis. Pak1‐mediated signaling enhances adaptive stress responses without substantial induction of pro‐apoptotic pathways. Activation of Pak1 with JB2019 mitigates cardiac dysfunction and cellular stress responses under metabolic stress, offering a promising therapeutic strategy. Targeting Pak1 and potentially developing isoform‐selective Pak modulators may provide a novel avenue for managing HFpEF, especially in metabolically compromised populations.

## Author Contributions

**Susanne S. Hille**: methodology, resources. **Hongyuan Zhang**: investigation, methodology, writing – review and editing, data curation, formal analysis, resources. **Yujia Cao**: investigation. **Bernard D. Keavney**: resources, methodology, investigation. **Yingjuan Liu**: methodology, investigation, resources. **He Yu**: resources. **Jiaqing Lang**: resources, methodology. **Honglin Xu**: conceptualization, investigation, writing – original draft, writing – review and editing, visualization, methodology, validation, software, formal analysis, project administration, data curation. **Ming Xu**: resources, conceptualization. **Min Zi**: investigation, methodology. **Elizabeth J. Cartwright**: resources, funding acquisition, methodology. **Oliver J. Müller**: methodology, resources. **Ming Lei**: methodology, resources. **Xin Wang**: conceptualization, funding acquisition, supervision, resources. **Tayyiba Azam**: conceptualization, methodology, data curation, investigation, validation.

## Conflicts of Interest

JB2019A was developed in Prof. Ming Lei's group at the University of Oxford and is covered in a pending United Kingdom Patent Application No. 2412195.6 owned by the University of Oxford. Tamer M. A. Mohamed holds equities in Tenaya Therapeutics. Tamer M. A. Mohamed is a co‐inventor on pending patents that relate to heart slice culture.

## Supporting information




**Supporting File 1**: advs76964‐sup‐0001‐SuppMat.pdf.

**Supporting File 2**: advs76964‐sup‐0002‐TableS1.pdf.

**Supporting File 3**: advs76964‐sup‐0003‐SuppMat.zip.

## Data Availability

The data that support the findings of this study are available on request from the corresponding author. The data are not publicly available due to privacy or ethical restrictions.
