# Supplementary material for: A Novel Pak1 Activator Ameliorates ER Stress for HFpEF Therapy
Source: Adv Sci (Weinh). 2026 Aug 3;13(60):e76964. doi: 10.1002/advs.76964 (PMC13430621; doi:10.1002/advs.76964)
Supplement: Supplementary file 2 [file ADVS-13-e76964-s003.pdf]

Supplementary 1

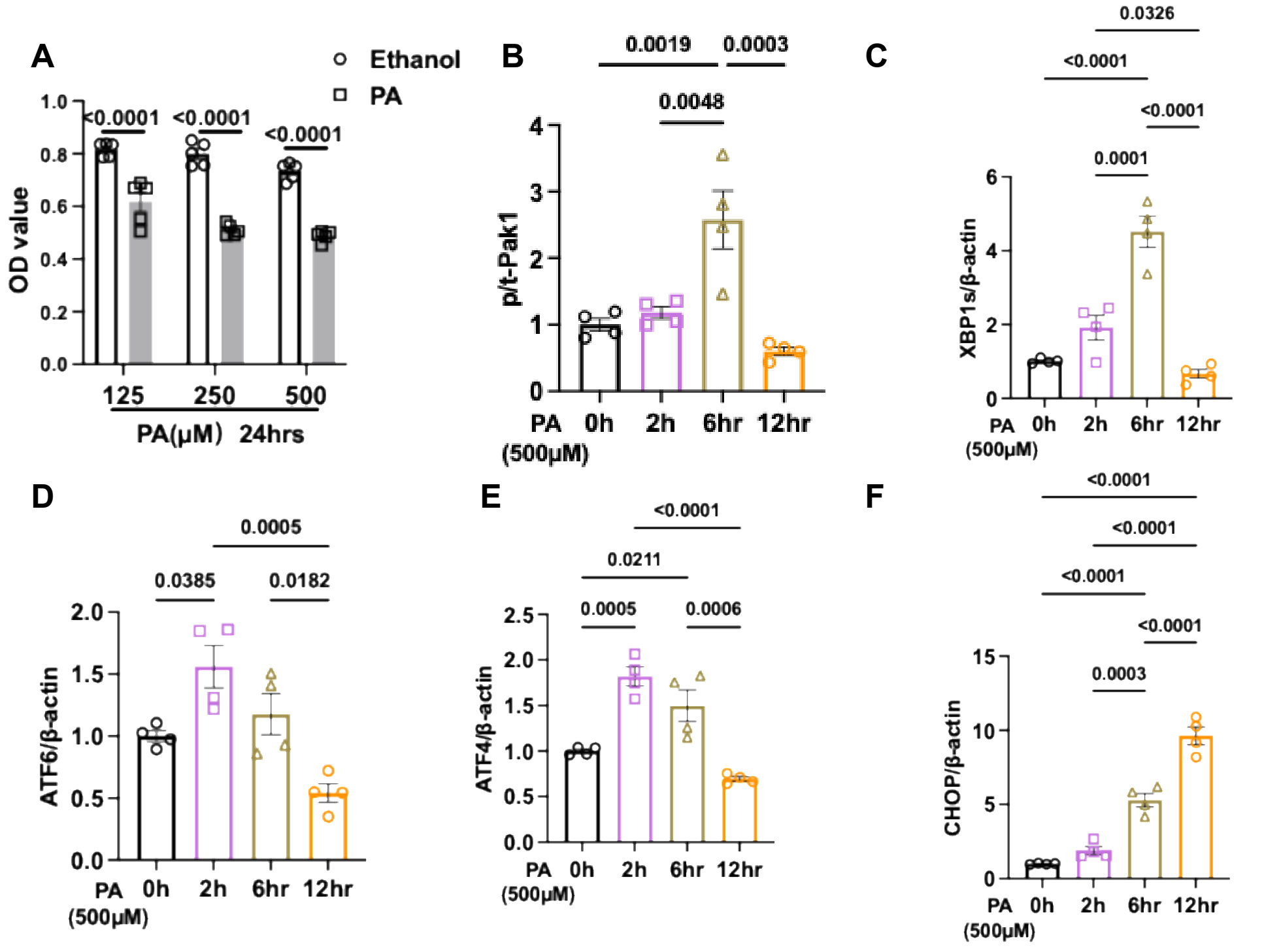

Figure S1: (A) Cytotoxicity by PA treatment (24 h) was evaluated by MTT assay in H9c2 cells. n = 5 per group. **(B-F)** Protein expression quantification of phosphorylated/total Pak1 (p/t-Pak1) and UPR related proteins in primary mouse cardiomyocytes. n = 4 per group. Data are presented as mean ± S.E.M. Statistical analysis was by one-way ANOVA followed by Tukey's multiple comparisons test.

Supplementary 2

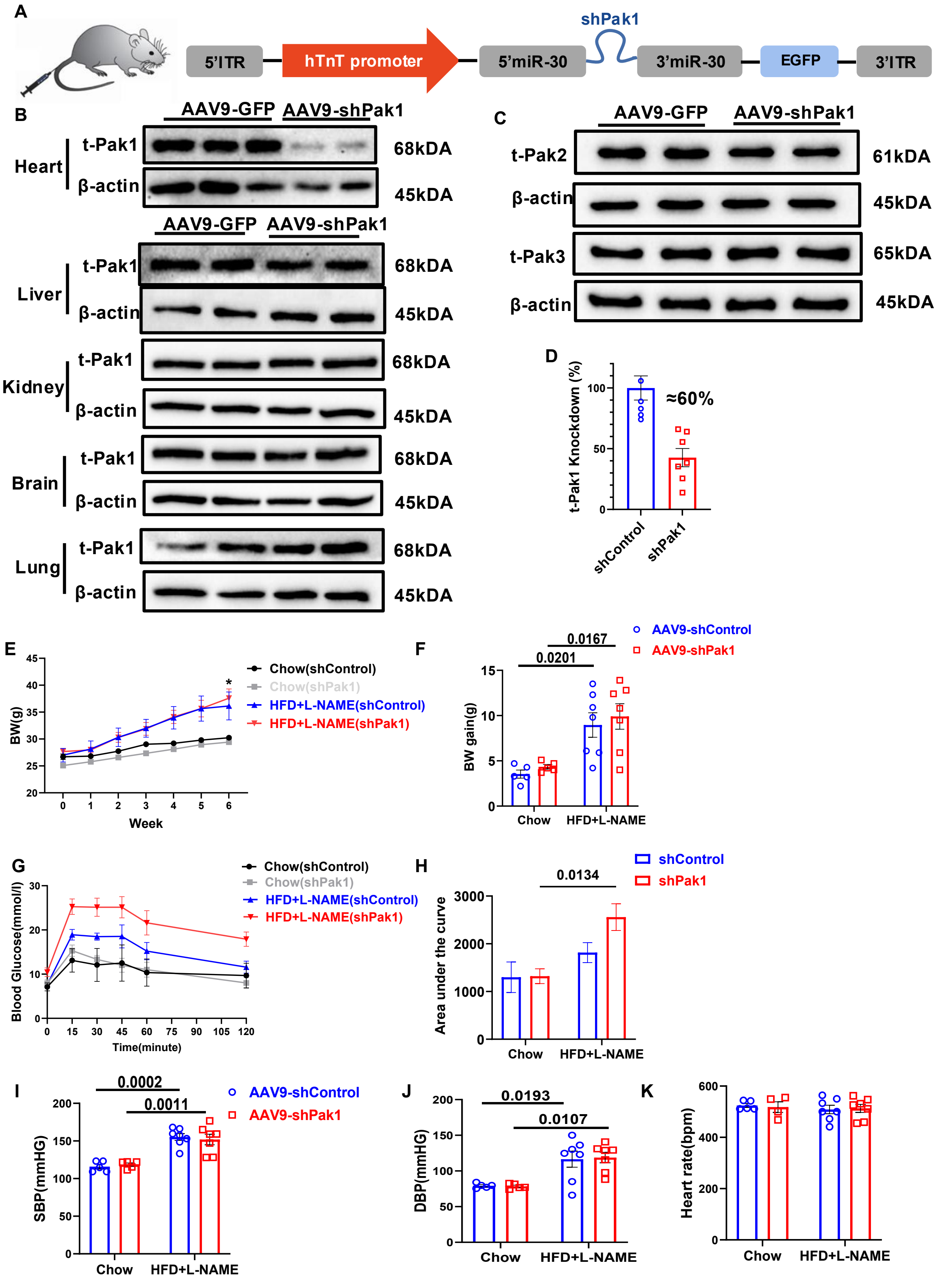

Figure S2: Cardiac-specific knockdown of Pak1 using AAV9 virus. (A) Schematic diagram showing AAV9 delivery via intravenous injection. **(B)** Pak1 expression levels in different tissues. **(C)** Pak2 and Pak3 expression in the heart. **(D)** Quantification of cardiac Pak1 expression in AAV-shPak1 mice compared with AAV-GFP controls. **(E)** Weekly body weight. **(F)** Body weight gain after treatment. **(G)** Intraperitoneal glucose tolerance test (IGTT) after 6 weeks of HFD+L-NAME treatment. **(H)** Bar graph showing the area under the curve (AUC) for IGTT. **(I and J)** Systolic and diastolic blood pressure after 6 weeks of HFD+L-NAME treatment. **(K)** Heart rate after 6 weeks of HFD+L-NAME treatment. Data are presented as mean ± SEM; n = 5–7 per group. Statistical analysis was performed using an unpaired two-tailed Student's t-test for panel D, two-way repeated-measures ANOVA for panels E, and two-way ANOVA for panels F and H–K. $^{*}P < 0.05$, HFD+L-NAME (shPak1) versus Chow (shPak1).

Supplementary 3

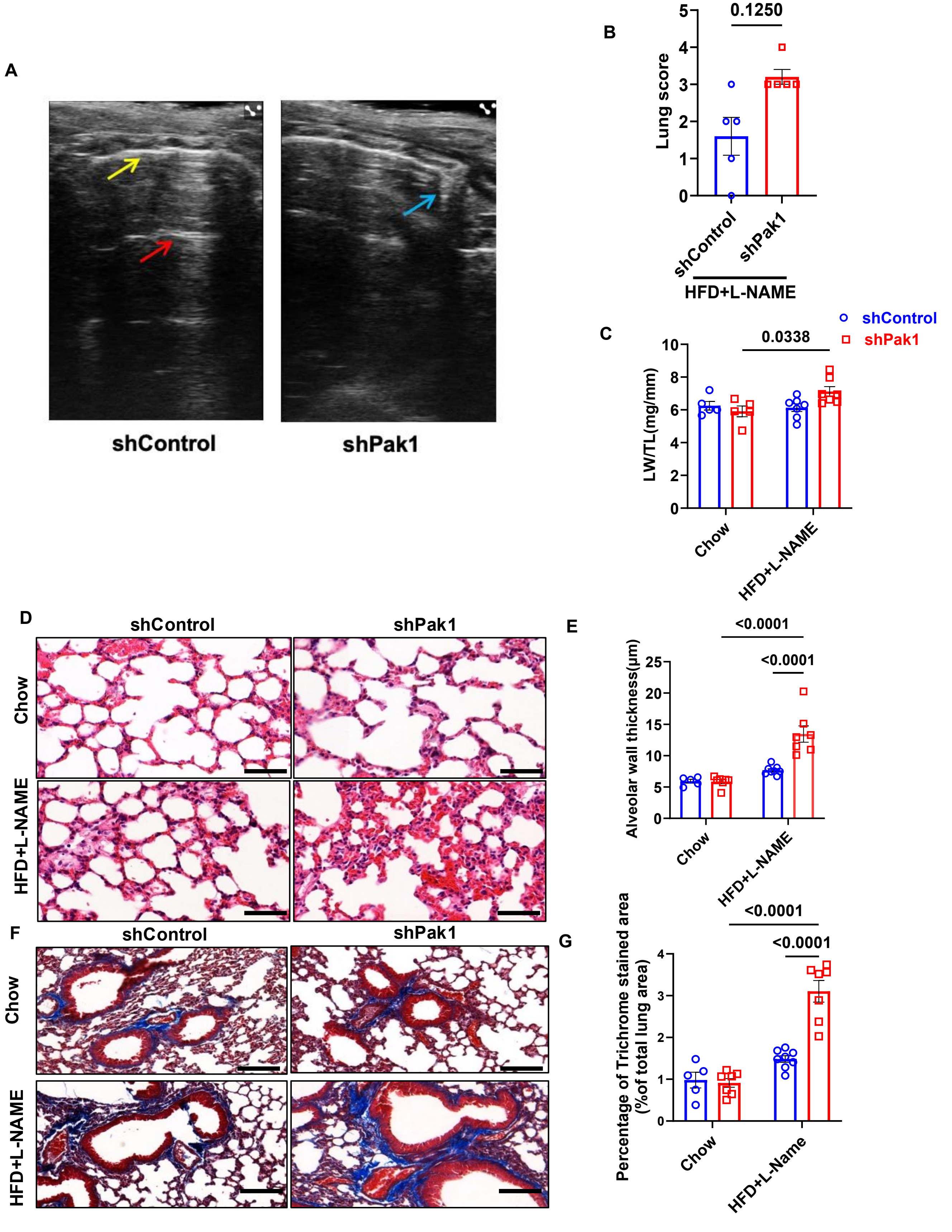

Figure S3: Knockdown of Pak1 in the heart induced lung injury after 6 weeks HFD+L-NAME treatment. (A) Lung ultrasound images. Yellow arrow showed thick pleural line and red arrow refered to A-lines, blue arrow refered to B-lines. **(B-C)** Lung ultrasound score and lung weight to tibia length ratio. **(D)** Representative images of H&E staining and **(F)** Masson's trichrome of lung. Scale bars: 50 μm (H&E) and 100 μm (Masson). **(E)** Thickness of alveolar wall. **(G)** Percentage of lung fibrosis area. n = 5 to 8 per group. Data are presented as means ± SEM. B, Wilcoxon matched-pairs signed-rank test; C, E and G, two-way ANOVA followed by Tukey's multiple comparisons test.

Supplementary 4

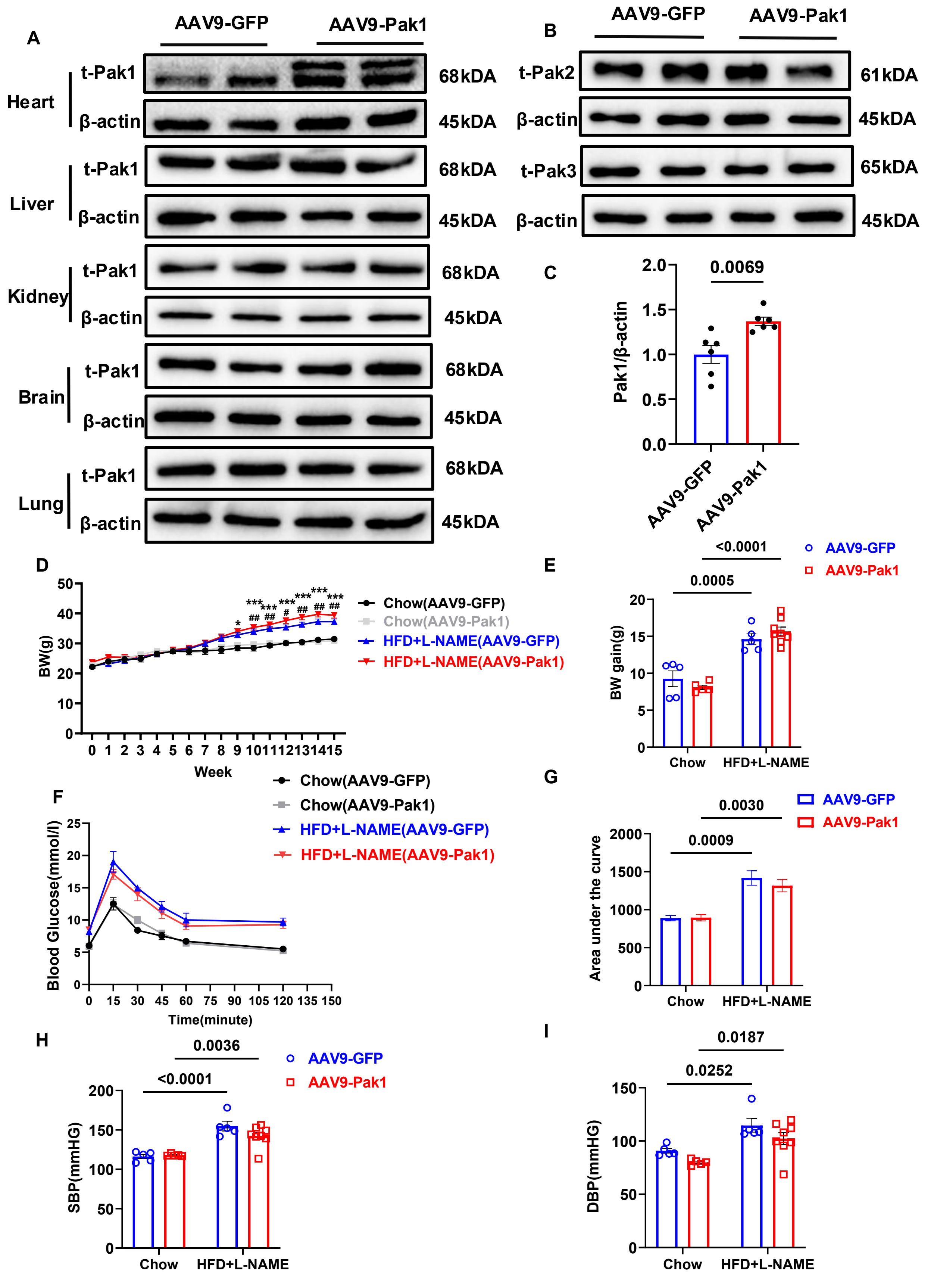

Figure S4: Cardiac-specific over-expression of Pak1 using AAV9 virus. (A) Pak1 expression levels in different tissues were detected by Western blotting. **(B)** Pak2 and Pak3 expression in the heart. **(C)** Quantification of cardiac Pak1 expression shown in panel A. n = 3 per group. **(D)** Weekly body weight. **(E)** Body weight gain, calculated as the difference in body weight between the end of treatment at 15 weeks and the beginning of the diet. **(F and G)** Intraperitoneal glucose tolerance test and corresponding area under the curve (AUC). **(H and I)** Systolic and diastolic blood pressure in different groups. Data are presented as mean ± SEM; n = 5-8 per groups. Statistical analysis was performed using two-way repeated-measures ANOVA for panel D and two-way ANOVA followed by Tukey's multiple comparisons test for panels E–I. $^{*}P < 0.05$ and $^{***}P < 0.001$ versus AAV9-Pak1 chow; $^{\#}P < 0.05$ and $^{\#\#}P < 0.01$ versus AAV9-GFP chow.

Supplementary 5

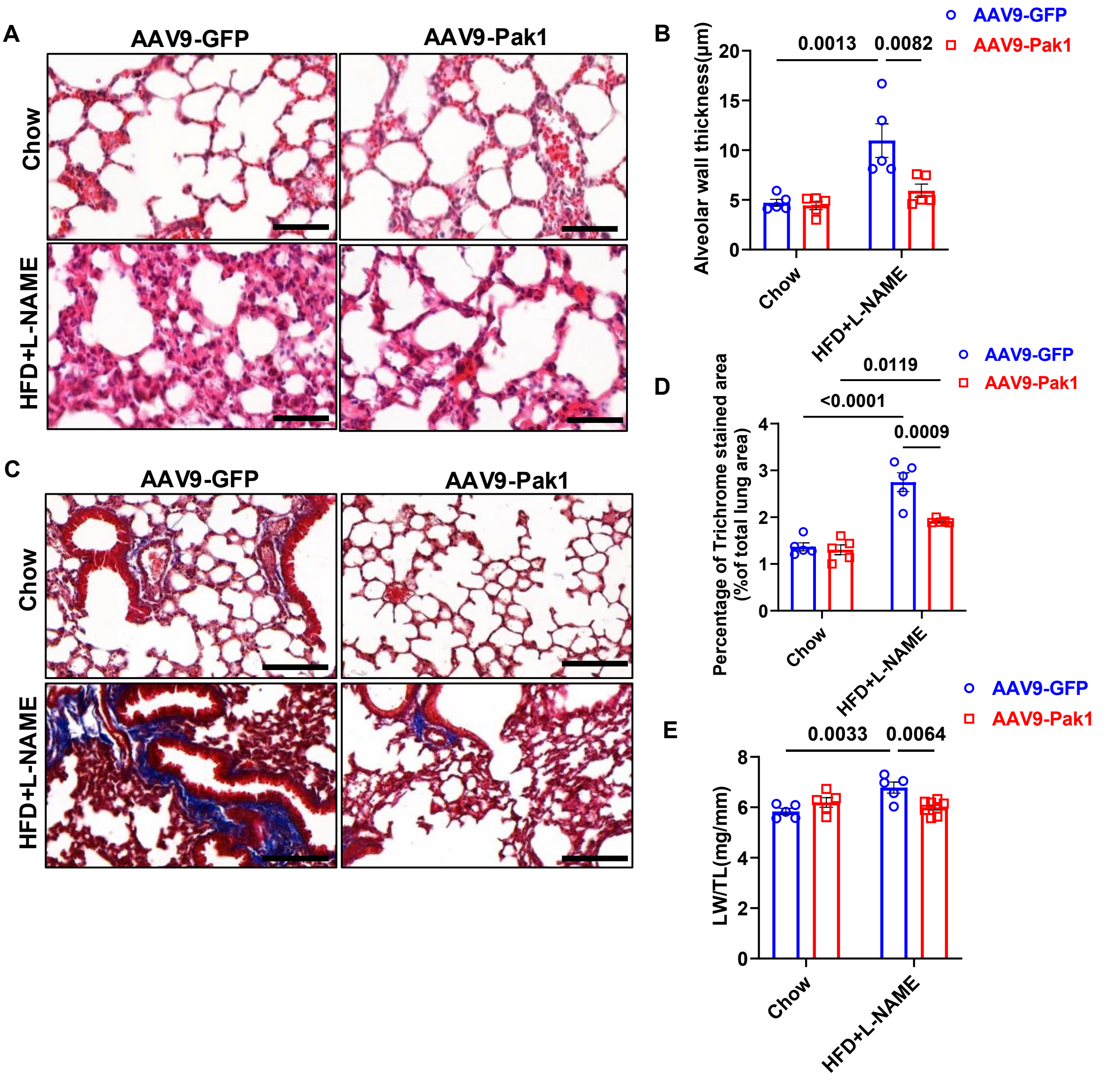

Figure S5: The effect of Pak1 over-expression on lung histology outcome following HFD+L-NAME. (A) Representative images of H&E staining and **(C)** Masson's trichrome of lung. Scale bars: 50μm (H&E) and 100μm (Masson). **(B)** Thickness of alveolar wall. **(D)** Percentage of lung fibrosis area. **(E)** Lung weight to tibia length ratio. n = 5 to 8 per group. Data are presented as means ± SEM. Statistical analysis was by two-way ANOVA followed by Tukey's multiple comparisons test.

Supplementary 6

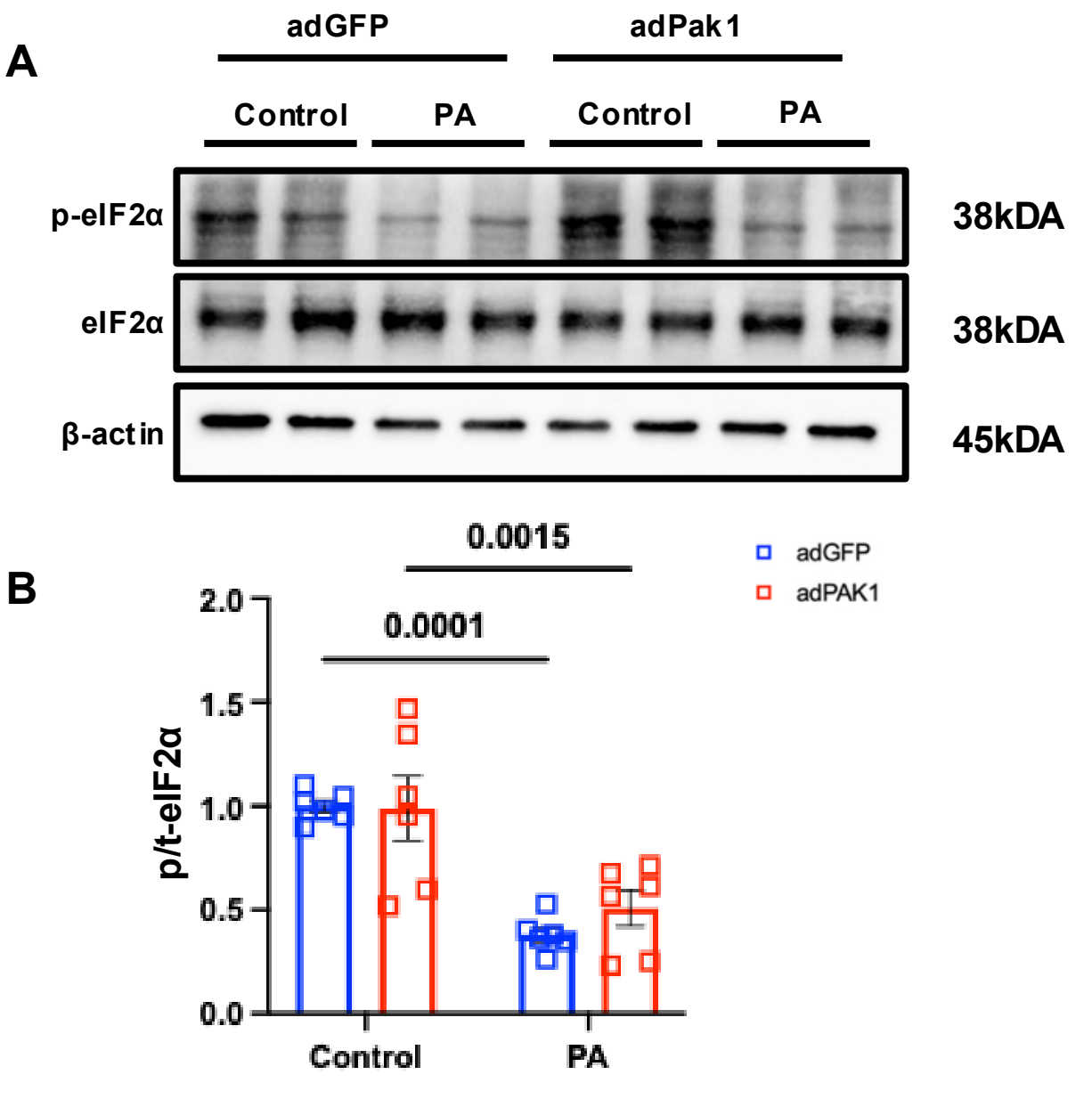

Figure S6: (A) Representative western blots of p/t-eiF2α. **(B)** Protein expression quantification of phosphorylated/total eIF2α. n = 6 per group. Data are presented as means ± SEM. Statistical analysis was by two-way ANOVA followed by Tukey's multiple comparisons test.

Supplementary 7

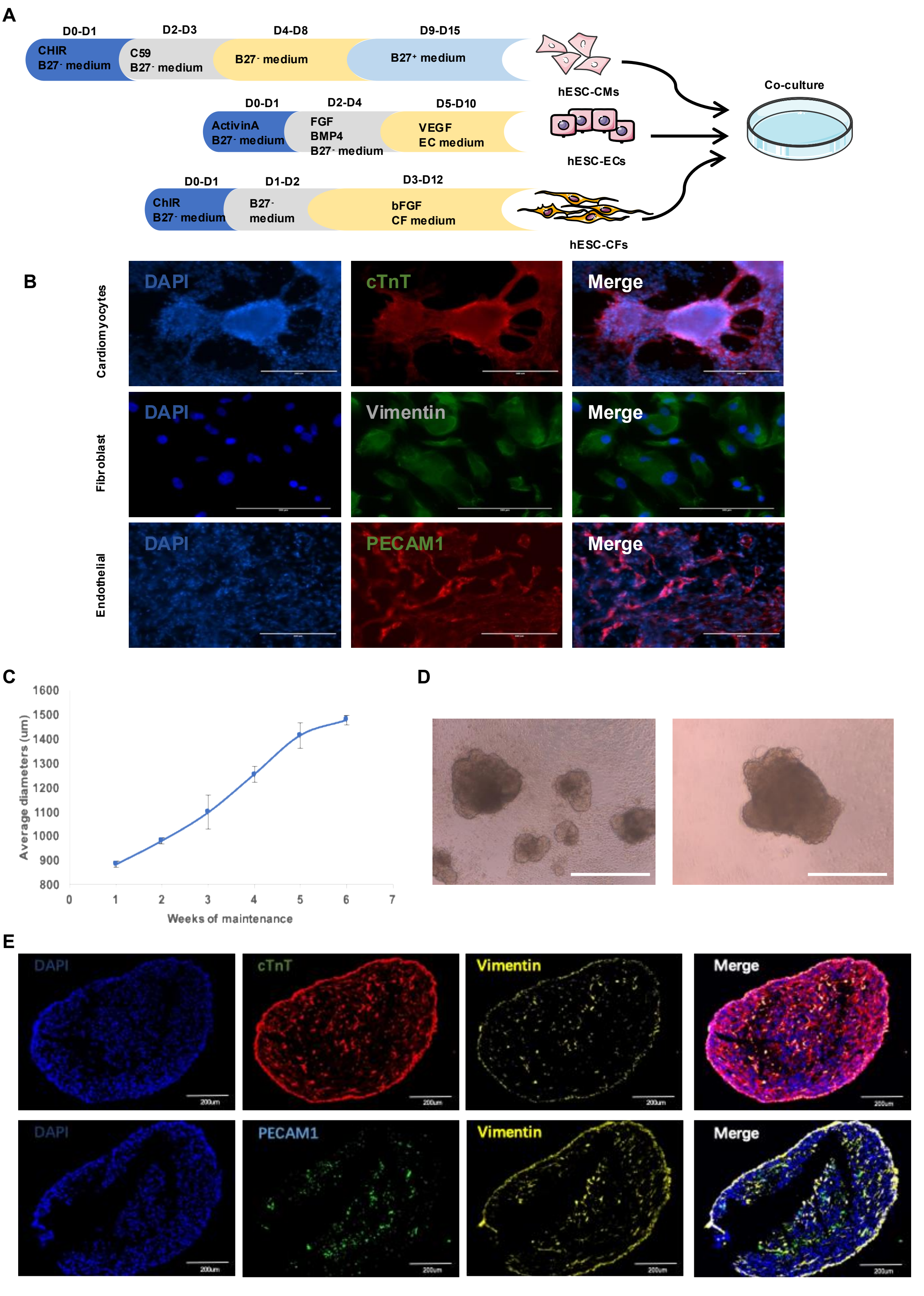

Figure S7: Cardiac differentiation of hESC and characterisation. (A) Cardiac differentiation timeline to generate cardiac organoids. **(B)** Immunofluorescent staining images of cardiomyocytes (cTnT), fibroblast (Vimentin) and endothelial cells (PECAM1) derived from hESC, scale bar = 200 μm. **(C)** Average diameters of cardiac organoids. **(D)** Image of cardiac organoids from day7 (left) and day21 (right), scale bar = 1000 μm. **(E)** Immunofluorescent staining images of human cardiac organoid, scale bar = 200 μm.

Supplementary 8

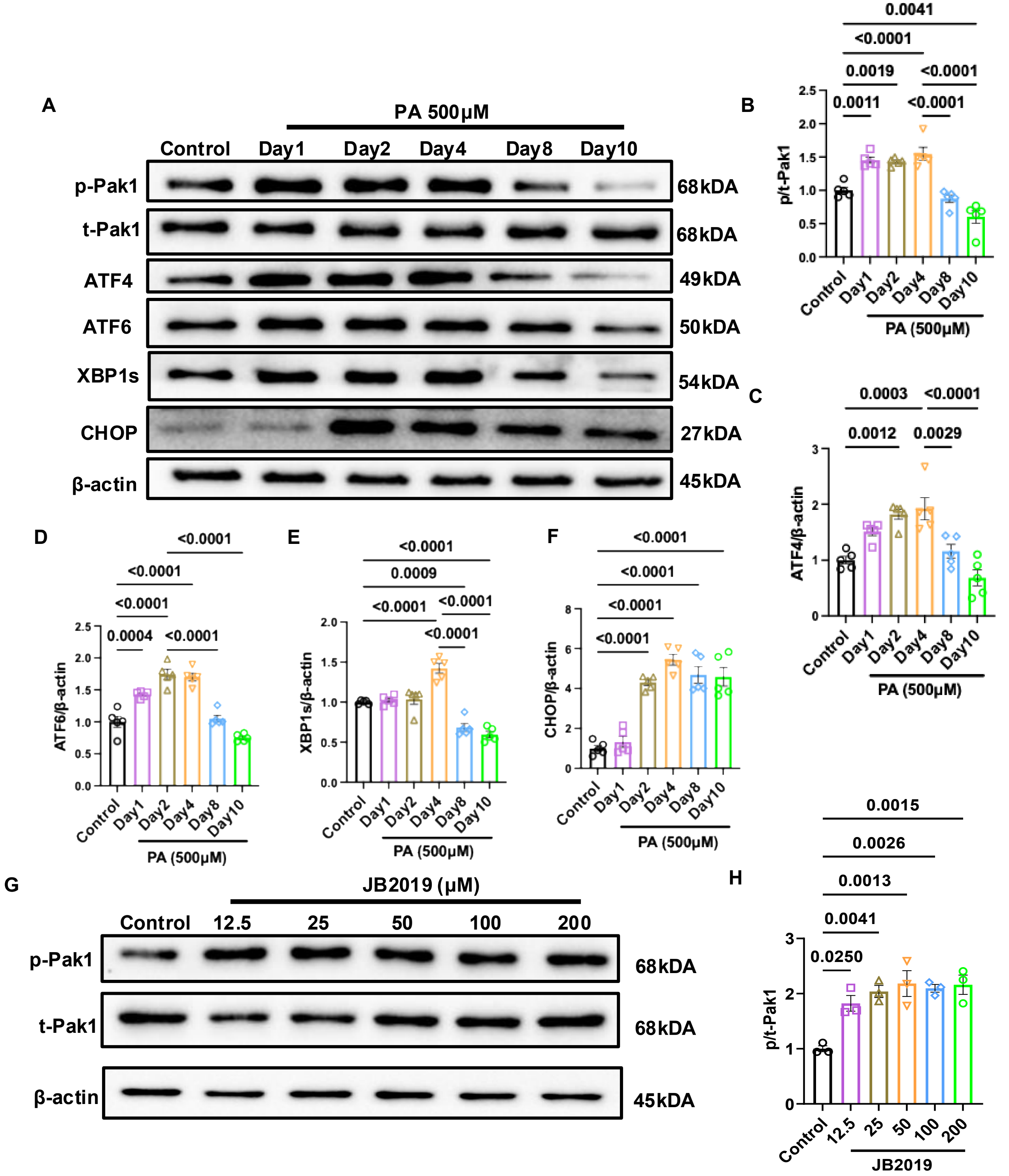

Figure S8: PA and compound JB2019 treatment in cardiac organoids. (A) Representative western blots of p/t-Pak1 and UPR pathway proteins and **(B-F)** Quantification of panel (A). n = 5 per group. **(G)** The Pak1 activation effect of JB2019 at different concentration. **(H)** Quantification of panel (G). n = 3 per group. Data are presented as means ± SEM. Statistical analysis was by two-way ANOVA followed by Tukey's multiple comparisons test.
