# Supplementary material for: A Novel Pak1 Activator Ameliorates ER Stress for HFpEF Therapy
Source: Adv Sci (Weinh). 2026 Aug 3:e76964. Online ahead of print. doi: 10.1002/advs.76964 (PMC13430621; doi:10.1002/advs.76964)
Supplement: Supplementary file 2 — Supporting File 2: advs76964‐sup‐0002‐TableS1.pdf. [file ADVS-9999-e76964-s002.pdf]

Table S1: List of Primary and Secondary Antibodies

Antibody	Manufacturer	Dilution
p-Pak1	Cell Signalling, # 2601S	1:1000
t-Pak1	Cell Signalling, # 2602S	1:1000
Pak2	Cell Signalling, #2608S	1:1000
Pak3	Cell Signalling, #2609S	1:1000
p-PERK	Abcam, ab192591	1:1000
PERK	Cell Signalling, # 3192S	1:1000
Cleaved-ATF6	Abcam, ab37149	1:1000
ATF4	Proteintech, 10835-1-AP	1:1000
XBP1s	Proteintech, 24868-1-AP	1:1000
CHOP	Cell Signalling, # 2895S	1:1000
P-eIF2α	Cell Signalling, # 9721S	1:1000
t-eIF2α	Cell Signalling, # 9722S	1:1000
P-eIF4e	Cell Signalling, # 9741S	1:1000
T-eIF4e	Cell Signalling, # 2067S	1:1000
P-eIF4BP1	Cell Signalling, # 9455S	1:1000
T-eIF4eBP1	Cell Signalling, # 9644S	1:1000
P-ERK1/2	Cell Signalling, # 4370S	1:1000
T-ERK1/2	Cell Signalling, # 9102S	1:1000
P-MNK1	Cell Signalling, # 2111S	1:1000
T-MNK1	Cell Signalling, # 2195S	1:1000
Anti-puromycin	Merck, MABE343	1:10000 for WB<br>1:1000 for IF
Calnexin	Cell Signalling, # 2679S	1:500 for IF
β-actin	Cell Signalling, # 4970S	1:1000
Gβ	Santa Cruz Biotechnology, sc-166123	1:1000
Monoclonal ANTI-FLAG® M2 antibody	Sigma-Aldrich, F1804	1:1000
Anti-Rabbit IgG HRP-linked antibody	Cell Signalling, # 7074S	1:6000
Anti-Mouse IgG HRP-linked antibody	Cell Signalling, # 7076S	1:6000
Anti-human CD31 (PECAM1)	Dako Omnis, GA610	1:20
Alexa Fluor® 647 Anti-Cardiac Troponin T antibody	Abcam, ab314677	1:200
Vimentin	Thermo Fisher Scientific, MA5-16409	1:100
